# Gaining insights into MmpL3: combining structural and computational approaches to unlock transport and inhibitor-binding mechanisms

**DOI:** 10.1107/S2059798326003050

**Published:** 2026-04-29

**Authors:** Satoshi Murakami, Domenico Marson, Eiki Yamashita, Bruno Broshka, Ui Okada, Maho Aoki, Giannamaria Annunziato, Erik Laurini, Emanuele Carosati, Marco Pieroni

**Affiliations:** a Department of Life Science and Technology, Institute of Science Tokyo, Yokohama226-8501, Japan; b Molecular Biology and Nanotechnology Laboratory (MolBNL@UniTS), Department of Engineering and Architecture (DEA) University of Trieste 34127Trieste Italy; c Institute for Protein Research Osaka University Suita565-0871 Japan; d Department of Chemical and Pharmaceutical Science University of Trieste 34127Trieste Italy; e Department of Food and Drug University of Parma 43124Parma Italy; European Bioinformatics Institute, United Kingdom

**Keywords:** MmpL3, indolecarboxamides, crystal structure, HPC molecular simulations, enhanced sampling MD

## Abstract

The rise of antimicrobial resistance in *Mycobacterium tuberculosis* underscores the urgent need for novel therapeutic strategies. Mycobacterial membrane protein large 3 (MmpL3) has emerged as a promising drug target, given its essential role in trehalose monomycolate transport and cell-wall biosynthesis. We determined the crystal structure of *M. smegmatis* MmpL3 in complex with a potent indolecarboxamide inhibitor, UPAR-1109, at 2.15 Å resolution, the highest reported to date. This structure provided unprecedented insights into the binding mode of the inhibitor, highlighting strong polar interactions and extensive hydrophobic contacts, which disrupt proton translocation. Speculative analysis about the molecular mechanism of inhibition has been proposed based on this model. Computational studies, including docking, molecular-dynamics and enhanced sampling simulations, revealed the remarkable plasticity of the MmpL3 binding site and confirmed the crystallographic orientation of UPAR-1109 as the most stable and biologically relevant binding mode. Together, these findings advance our understanding of the function and inhibition of MmpL3, providing valuable information for the rational design of next-generation antituberculosis agents, also with potential applications against nontuberculous mycobacteria.

## Abstract 2

This study investigates the mycobacterial membrane protein MmpL3, a key target for new antituberculosis drugs, by combining high-resolution crystallography and computational simulations. It reveals how the inhibitor UPAR-1109 binds to MmpL3, providing detailed insights into the function and flexibility of the protein and supporting the rational design of next-generation antimycobacterial therapies.

## 1. Introduction

The rise of antimicrobial resistance (AMR) poses a significant threat to global public health. Untreated resistant infections are estimated to surpass cancer as a cause of death by 2050 (Naghavi *et al.*, 2024 ▸). Among the bacterial pathogens that are most affected, *Mycobacterium tuberculosis* (*Mtb*), the causative agent of tuberculosis (TB), is of particular concern (Global Tuberculosis Programme, World Health Organization; https://www.who.int/teams/global-tuberculosis-programme/diagnosis-treatment/treatment-of-drug-resistant-tb/types-of-tb-drug-resistance). Despite advances in healthcare, TB remains a major global health issue, with an estimated one-third of the world’s population infected, and approximately 10.8 million new cases and 1.25 million deaths in 2023 (World Health Organization, 2024*a* ▸). The current multi-drug regimen, which requires up to nine months of simultaneous administration of four antibiotics (rifampin, isoniazid, ethambutol and pyrazinamide), negatively impacts patient adherence and treatment effectiveness. Besides, multidrug-resistant (MDR-TB) and extensively drug-resistant (XDR-TB) strains have emerged, further complicating treatment strategies (World Health Organization, 2024*b* ▸). In this scenario, the search for novel therapeutic targets is of the utmost importance.

Among these, mycobacterial membrane protein large 3 (MmpL3) has recently become the focus of intense research, with considerable effort dedicated to its structural characterization. MmpL3, one of several MmpL proteins encoded by the *Mtb* genome (Cole *et al.*, 1998 ▸; La Rosa *et al.*, 2012 ▸; Grzegorzewicz *et al.*, 2012 ▸), plays a critical role in *Mtb* pathogenesis. It is involved in the transport of trehalose monomycolate (TMM), an essential precursor for mycolic acid synthesis. MmpL3 is proposed to function as a TMM flippase (Degiacomi *et al.*, 2020 ▸; Xu *et al.*, 2017 ▸), utilizing a mechanism of active secondary transport to export mycolic acids. This process couples the transport of mycolic acids out of the cell with the inward flow of protons, driven by energy derived from the electrochemical gradient across the cell membrane, established by the bacterial metabolism. This transport, crucial for cell-wall biogenesis, makes MmpL3 an attractive target for novel therapies, particularly in the context of increasing drug resistance, with consequent interest in developing inhibitors (Williams & Abramovitch, 2023 ▸).

One of the most notable facts about MmpL3 is the number of diverse chemotypes that have been shown to inhibit *Mtb* growth by targeting this protein. These inhibitors have been identified both through whole-cell screening of compound libraries as well as through whole-genome sequencing of spontaneously resistant mutants. Examples include 1,5-diarylpyrroles such as BM212 and derivatives (La Rosa *et al.*, 2012 ▸; Poce *et al.*, 2013 ▸, 2016 ▸), the benzimidazole C215 (Stanley *et al.*, 2012 ▸), tetrahydropyrazolo[1,5-a]pyrimidine-3-carboxamides (THPPs; Remuiñán *et al.*, 2013 ▸), *N*-benzyl-6,7-dihydrospiro[piperidine-4,4-thieno[3,2-c]pyran]s (Remuiñán *et al.*, 2013 ▸), indolecarboxamides (Onajole *et al.*, 2013 ▸; Lun *et al.*, 2013 ▸; Rao *et al.*, 2013 ▸; Kondreddi *et al.*, 2013 ▸; Stec *et al.*, 2016 ▸), adamantyl ureas (AUs) such as AU1235 (Grzegorzewicz *et al.*, 2012 ▸), diamides such as SQ109 (Lee *et al.*, 2003 ▸; Nikonenko *et al.*, 2007 ▸; Sacksteder *et al.*, 2012 ▸; Tahlan *et al.*, 2012 ▸) and spiro-compounds such as SPIRO (Remuiñán *et al.*, 2013 ▸) and ST004 (Hu *et al.*, 2022 ▸), which combines SQ109 with rimonabant, a CB1 cannabinoid receptor antagonist identified as an antitubercular agent through drug repurposing (An *et al.*, 2020 ▸).

The promiscuous nature of MmpL3 as a drug target, possibly due to its inherent vulnerability or druggability (Li, Upadhyay *et al.*, 2014 ▸), has sparked the attention of TB scientists, especially taking into consideration the variety of chemical structures identified and their activity profiles. For instance, the broad antibacterial and antifungal activity of SQ109, BM212 and THPPs, including against organisms lacking mycolic acids, contrast with the mycobacteria-specific action of AUs and indolecarboxamides. In addition, these MmpL3 inhibitors differ in their activity against nonreplicating *M. tuberculosis*, with SQ109, BM212 and THPPs being effective against dormant bacilli, whereas AUs and indolecarboxamides lack activity towards these strains.

**Figure 1:**
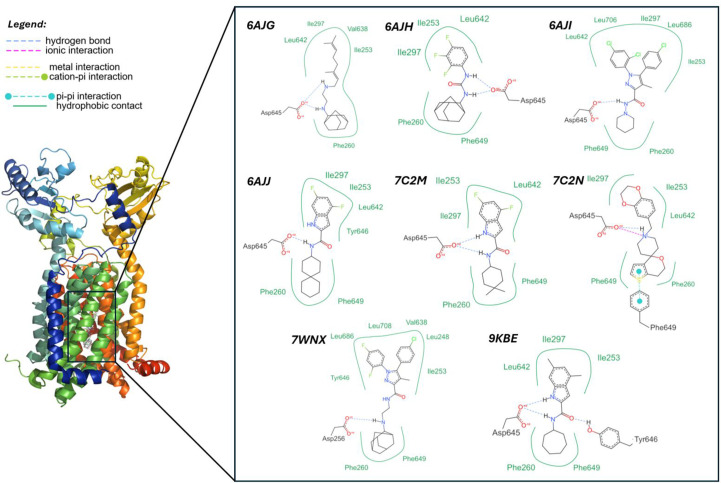
2D depiction of UPAR-1109 and other inhibitors in complex with MmpL3. The images represent the two-dimensional interaction maps of each ligand within its corresponding MmpL3 crystal structures, highlighting the key protein–ligand interactions. The interaction diagrams were generated using *ProteinPlus* (Ehrt *et al.*, 2025 ▸; https://proteins.plus/).

Among all of these structures (Fig. 1 ▸), indolecarboxamides represent a suitable pharmacological tool for further investigating the interaction of MmpL3 with its inhibitors. These molecules exhibit potent and selective activity against mycobacteria, including resistant strains. Moreover, their chemical accessibility makes them excellent candidates for further structural variation, so as to improve/modulate antibacterial potency and drug-like properties.

The first crystal structures of MmpL3, both in the apo form and with inhibitors, were reported in 2019 (Zhang *et al.*, 2019 ▸). The researchers used *M. smegmatis* for these initial studies, leveraging its ease of cultivation, faster growth and high structural homology to the *Mtb* ortholog. These initial structures provided insights into the transport mechanism of MmpL3 that are likely to be applicable to the *Mtb* protein, and indeed such homology was structurally confirmed a few years later (PDB entry 7nvh; Adams *et al.*, 2021 ▸). Similarly to other transport proteins (*i.e.* the bacterial multidrug-efflux transporter AcrB; Murakami *et al.*, 2002 ▸), MmpL3 consists of 12 helices in the transmembrane region, and two periplasmic subdomains (PD1 and PD2) which together form a central cavity that facilitates substrate transport.

Additional studies on *M. smegmatis*, through electron microscopy reconstituted into lipidic nanodiscs (Su *et al.*, 2019 ▸, 2021 ▸), have identified specific interactions between MmpL3 and various ligands, including phosphatidylethanolamine (PE) and glycolipids in the TMM binding region, whereas the first (and to date only) structural investigation of *Mtb* MmpL3 was carried out through crystallography and released in 2021 (Adams *et al.*, 2021 ▸). In particular, it was observed that the interactions with inhibitors involve hydrophilic and hydrophobic residues of the binding site, which stabilize ligand binding, hence providing insights into proton transport. The disruption of the hydrogen bond between Asp645 and Tyr257 and the hydrogen bond formed between Asp256 and Tyr646 are critical for MmpL3 inhibition, as these interactions are key to proton passage across the cell membrane (Alsayed *et al.*, 2021 ▸).

The distinct conformations observed by crystallography (X-ray) and electron microscopy (cryo-EM) (Couston *et al.*, 2023 ▸) highlight the dynamic nature of MmpL3. This conformational dynamism might suggest a functional link between conformational flexibility and transport function. Furthermore, this flexibility might allow the binding of a wide range of chemical pharmacophores, explaining the promiscuous nature of MmpL3 as a drug target. Despite these facts, all of the MmpL3 structures available to date in the Protein Data Bank archive (PDB; Berman *et al.*, 2000 ▸) have a resolution of 2.6 Å or worse (Table 1 ▸), thus affecting the quality and reliability of structure-based investigations. Moreover, the lack of comprehensive studies that explore the binding modes of MmpL3 inhibitors represents another significant gap in understanding those factors that significantly impacts the efficacy and specificity of potential therapeutic agents. In this regard, computational methods of various kinds can provide decisive support to the task.

Therefore, this study aims to employ a multidisciplinary approach to further characterize the structure of MmpL3 and its interactions with a suitable ligand. To achieve this, we utilized *N*-cycloheptyl-4,6-dimethyl-1H-indole-2-carboxamide (UPAR-1109; Onajole *et al.*, 2013 ▸) to obtain a high-resolution crystal structure. UPAR-1109 is a known potent indolecarboxamide with extraordinary bactericidal activity against *Mtb*, the alleged molecular target of which is MmpL3 (Lun *et al.*, 2013 ▸). This structure has served as a starting point for subsequent computational investigations and molecular-dynamics studies, delivering a deeper understanding of the structural characteristics of MmpL3, and ultimately paving the way for the development of additional series of specific inhibitors.

## 2. Experimental sections

### 2.1. Protein preparation

A gene encoding C-terminally truncated MmpL3 (1–773) from *M. smegmatis* was amplified from genomic DNA (mc^2^ 155) by polymerase chain reaction (PCR) using KOD-plus-Neo DNA polymerase (TOYOBO, Japan) with forward 5′-GTCGCTGCATATGTTCGCCTGGTGGGGTCGGACCGTGTAC-3′ and reverse 5′-CCGCTCGAGTCAGTGATGATGATGATGATGCCGCTGATCGGTCTCGGACTCGCGCACGGTG-3′ primers and inserted via NdeI and XhoI restriction sites into a pET-22b (Invitrogen) expression vector. Using this method, a C-terminal hexahistidine tag was added to aid in purification by immobilized metal-affinity chromatography. The DNA was sequenced to confirm the intended modifications. The resulting plasmid was transformed into *Escherichia coli* C43 (DE3) cells for protein expression. The transformants were grown in ten 5 l flasks at 25°C in Davis minimal medium (Davis, 1949 ▸) supplemented with 0.2%(*w*/*v*) glucose and 0.1%(*w*/*v*) casamino acids. Expression was induced for 8 h by adding 0.1 m*M* isopropyl β-d-1-thiogalactopyranoside (IPTG) at an OD_610 nm_ of 0.6. All subsequent procedures were performed at 4°C unless indicated otherwise. The cells were harvested by centrifugation, resuspended in 0.1 *M* sodium phosphate pH 7.2, 1 m*M* EDTA, 10%(*v*/*v*) glycerol and disrupted three times using a Microfluidizer M-110EH (Microfluidics, New Mexico, USA) at 103 MPa. Cell debris was removed by low-speed centrifugation at 27 000*g* for 10 min. To collect membrane fractions, the supernatant was subjected to ultracentrifugation at 145 000*g* for 1 h and washed with 20 m*M* sodium phosphate pH 7.2, 2 *M* KCl, 1 m*M* EDTA, 10%(*v*/*v*) glycerol. The plasma membrane was then solubilized in 20 m*M* 4-(2-hydroxyethyl)-1-piperazineethanesulfonic acid (HEPES) pH 7.5 containing protease inhibitors (Roche) and 2%(*w*/*v*) *n*-dodecyl-β-d-maltoside (LMT; Glycon Biochemicals GmbH, Germany) on ice for 1 h. After a further step of ultracentrifugation at 145 000*g* for 1 h, the detergent-solubilized fraction was harvested and incubated with Ni Sepharose at 4°C for 1 h. The resin was washed with buffer consisting of 20 m*M* HEPES pH 7.5, 150 m*M* NaCl, 60 m*M* imidazole, 0.05%(*w*/*v*) LMT. The protein was eluted from the affinity resin with the wash buffer plus 300 m*M* imidazole. Fractions containing MmpL3 were collected, concentrated in an Amicon Stirred Cell (Merck Millipore) with a 100 kDa molecular-weight cutoff Omega Ultrafiltration Membrane Disk Filter (Pall Corporation, USA) and filtered with an Ultrafree-MC GV Centrifugal Filter (Merck Millipore). Further purification was performed by size-exclusion chromatography (Superdex-200 Increase 10/300 GL; Cytiva) in buffer consisting of 20 m*M* HEPES pH 7.5, 150 m*M* NaCl, 0.05%(*w*/*v*) LMT at a flow rate of 0.3 ml min^−1^ using an ÄKTAexplorer 10S (Cytiva). The peak fractions were collected and concentrated in the same way as described above to ∼25 mg ml^−1^ membrane protein for crystallization.

### 2.2. Crystallization of MmpL3 in complex with UPAR-1109

Purified MmpL3 (234 µ*M*) was mixed with UPAR-1109 (1.17 m*M*) and incubated for 12 h. MmpL3–UPAR-1109 complex crystals were grown by the sitting-drop vapor-diffusion technique at 25°C. Protein solution was mixed (1:1) with reservoir solution consisting of 31–33% polyethylene glycol (PEG) 400, 50 m*M* magnesium acetate, 100 m*M* sodium acetate pH 5.1. Crystals grew within 2–3 weeks to optimal size (0.3 × 0.2 × 0.2 mm). The concentration of PEG 400 was gradually increased to 38%(*v*/*v*) by soaking for optimal cryoprotection. Crystals were picked up using nylon loops (Hampton Research, California, USA) for flash-cooling in cold nitrogen gas from a cryostat (Rigaku, Japan).

### 2.3. Crystallographic data collection and structure determination

Data sets were collected at 100 K using an EIGER hybrid photon-counting (HPC) pixel-array detector (Dectris, Switzerland) on the BL44XU beamline at SPring-8. Diffraction images were processed with the *XDS* package (Kabsch, 2010*a* ▸,*b* ▸). Further processing was carried out with programs from the *CCP*4 suite (Agirre *et al.*, 2023 ▸) and *Phenix* (Adams *et al.*, 2002 ▸, 2010 ▸; Liebschner *et al.*, 2019 ▸). Data-collection and structure-refinement statistics are summarized in Table 2 ▸. The crystal structure was solved by the molecular-replacement method using *Phaser* (McCoy *et al.*, 2007 ▸) with the MmpL3 structure (PDB entry 6or2; Eicher *et al.*, 2012 ▸) as the search model. Model building was carried out using *Coot* (Emsley & Cowtan, 2004 ▸). Model refinement was conducted using *phenix.refine* (Adams *et al.*, 2002 ▸, 2010 ▸). Ramachandran analysis revealed 98.9% of residues in the favored region and 0% as outliers, with a *MolProbity* (Chen *et al.*, 2010 ▸) score of 1.54. In all of the sections of the manuscript, the figures were prepared using *PyMOL* (https://www.pymol.org) or *UCSF Chimera* (Pettersen *et al.*, 2004 ▸).

## 3. Computational studies

### 3.1. Protein alignment, pocket identification and characterization

We searched the PDB for MmpL3, and filtered the results by selecting only those PDB entries complexed with an inhibitor: six structures were from crystallographic data [PDB entries 6ajg, 6ajh, 6aji, 6ajj (Zhang *et al.*, 2019 ▸), 7c2m and 7c2n (Yang *et al.*, 2020 ▸)] and one was from electron microscopy (PDB entry 7wnx; Hu *et al.*, 2022 ▸), with a higher resolution of 3.36 Å. We extended the analysis to the structure complexed with UPAR-1109 (PDB entry 9kbe) obtained using the methods detailed above. As a preliminary step, we used *PyMOL* to align the coordinates and to adjust the input for further steps (ligand extraction and addition of H atoms to polar atoms, both on the protein and the ligands). Subsequently, the *BioGPS* software (Siragusa *et al.*, 2015 ▸, 2016 ▸) was used to identify the pocket of interest (containing the inhibitor) by setting the parameters sensitivity to 3 (the default is 8) and erosion to 3 (the default is 3); this, for all of the entries, led to the identification of pockets characterized by regular shape, without fringed margins, but allowing pocket extension wherever possible. Finally, we used *GRID* molecular inter­action fields (MIFs) to characterize the MmpL3 binding site through hotspot identification; for this purpose, we used the *GRID* hydrophobic probe DRY, as well as the polar *GRID* probes N1 and O, to detect favorable regions for interacting with hydrogen-bond donating and accepting groups, respectively. We also used the probe OH2 to detect favorable hotspots for water molecules.

### 3.2. Study of flexibility

To analyze the flexibility of residues in the MmpL3 binding pocket, a Python script was developed utilizing functions from the Biopython library (Cock *et al.*, 2009 ▸). The script calculates the mean pairwise distances between the atoms of each residue, accounting for peculiarities arising from the symmetric geometry of certain side chains. The analysis was performed on 20 amino acids constituting the pocket, with a score (referred to below as ‘flexibility index’) assigned to each residue based on an equation that reflects the root-mean-square distance (r.m.s.d.). Since r.m.s.d. typically applies to pairs of structures, we exhaustively combined the seven structures in pairs, and the final r.m.s.d. average constituted the flexibility index reported in the analysis. Residues of the binding pocket were classified as either ‘flexible’, ‘moderately flexible’ or ‘fixed’, based on the comparison of their values with thresholds derived from the mean and standard deviation of the flexibility index distribution.

### 3.3. Redocking

Redocking experiments (Bender *et al.*, 2021 ▸) were based on the software *AutoDock Vina* (Eberhardt *et al.*, 2021 ▸; Trott & Olson, 2010 ▸). The 3D coordinates of the MmpL3 inhibitors were exported as SDF files from the corresponding PDB files using *PyMOL*. Several inhibitors were docked versus their corresponding complexes and the best docking poses were compared with the experimental data through r.m.s.d. values to numerically assess the quality of the redocking solutions. Docking parameters were set as follows: energy_range = 3 (as the default), exhaustiveness = 32 (the default is set to 8) and num_modes = 10 (as the default). Finally, three docking runs varied in the rigid/flexible docking setup and the size of the grid cage; thus, different runs were named rsg (with r standing for rigid and sg for small grid), rlg (with lg standing for large grid) and fsg (with the prefix f standing for flexible docking). For each complex, the center of the cage was set to (*x* = 14.7, *y* = 17.55, *z* = 32.90), coordinates derived from the barycenter of the pocket identified by *BioGPS*, and previously aligned on the structure 7c2n. Instead, the size of the grid was defined as follows: for the small grid, containing only the central portion of the pocket, the size was set to 20.25 Å (on the *x* axis), 23.25 Å (on the *y* axis) and 23.25 Å (on the *z* axis) and for the large grid, which included an extended volume through the cytoplasm entry as evident in 9kbe, the size was set to 20.25 Å (on the *x* axis), 28.50 Å (on the *y* axis) and 37.50 Å (on the *z* axis).

### 3.4. Unbiased molecular dynamics (uMD)

The crystal structure of MmpL3 in complex with UPAR-1109 from this work was taken as the starting configuration. The physiological protonation states of the relevant residues were assigned using the *H*++ webserver (Anandakrishnan *et al.*, 2012 ▸). Subsequently, the final parametrization of MmpL3 was achieved with the AMBER ff14SB force field (Maier *et al.*, 2015 ▸). Atom types, as per the GAFF2 force field (Wang *et al.*, 2004 ▸), were assigned to the drug through the *antechamber* module of *AMBER* 2024 (Case *et al.*, 2023 ▸). The partial charges of the drug atoms were computed by the *RED* server (Vanquelef *et al.*, 2011 ▸), following the restrained electrostatic potential method. The *tleap* module of *AMBER* 2024 was used to solvate the complexes with TIP3P water molecules (Jorgensen *et al.*, 1983 ▸; allowing at least 15 Å from the solute and the box edges) and to add the proper number of sodium and chlorine ions to achieve a neutral system. The final topology obtained from *tleap* was converted to a *GROMACS*-compatible topology via the *parmed* tool (Shirts *et al.*, 2017 ▸), which was used for the enhanced sampling simulations.

**Figure 2:**
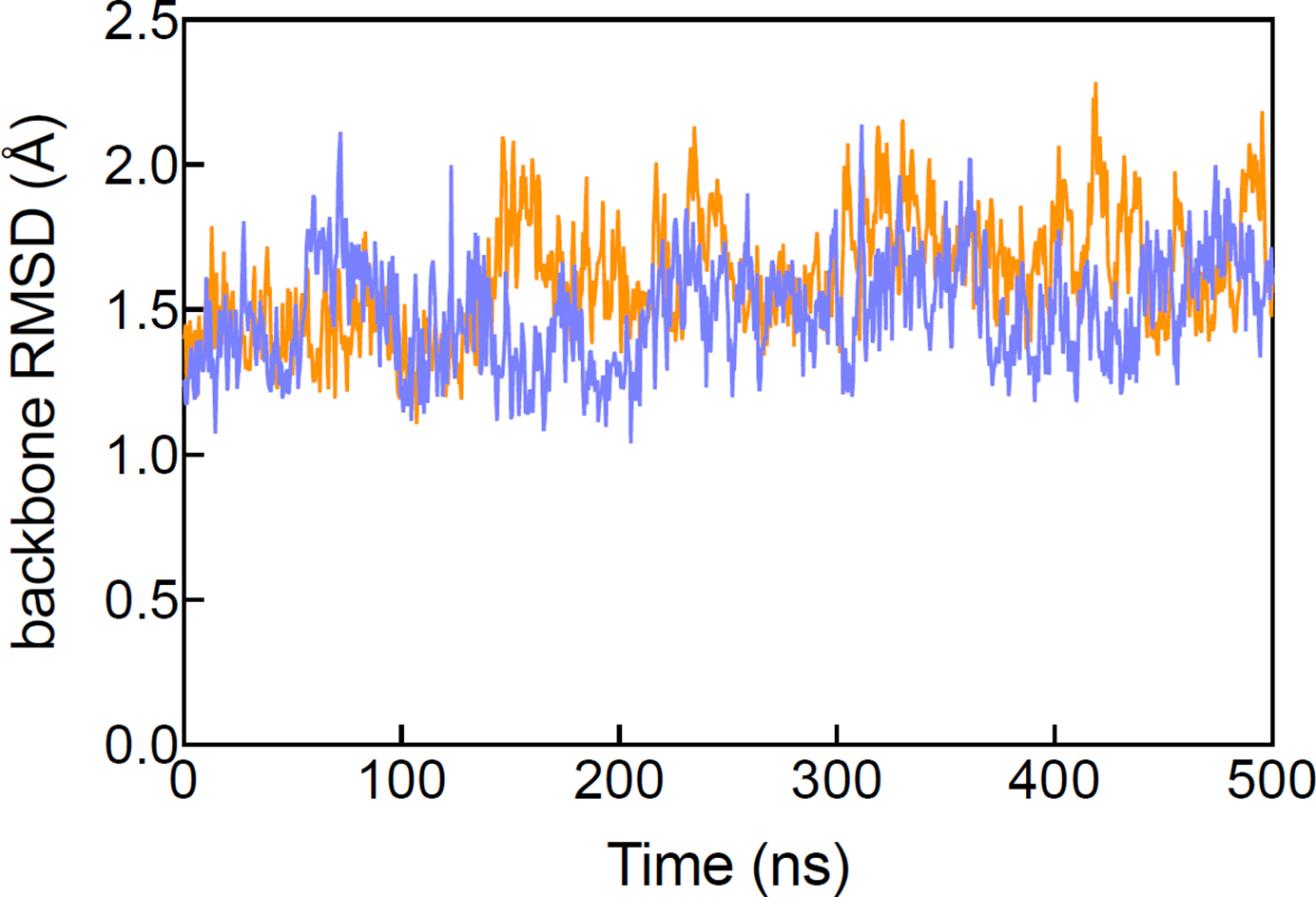
Backbone r.m.s.d. (Å) of MmpL3 in complex with UPAR-1109 (purple, crystal structure; orange, reversed orientation) as a function of uMD simulation time, computed with respect to the crystallographic structure.

MD simulations were performed in aqueous solution without an explicit lipid bilayer. This simplified environment was chosen to (i) keep the overall enhanced-sampling campaign computationally tractable [multiple long uMD trajectories, many random-accelerated molecular-dynamics (raMD) replicas and multiple-walker on-the-fly probability enhanced sampling (OPES)] and (ii) ensure a consistent, like-for-like comparison between alternative ligand orientations starting from the same crystallographic framework. Importantly, fold integrity in this aqueous setup was monitored *a posteriori* by computing the protein backbone r.m.s.d. relative to the crystallographic structure throughout the uMD trajectories (reported in Fig. 2 ▸).

A well established protocol (Russi *et al.*, 2022 ▸) includes drug–protein complex equilibration followed by 500 ns of unrestrained and unbiased molecular dynamics (conventional, *i.e.* without any added bias potential, uMD) simulation. Precisely, the simulation box was initially subjected to energy minimization, where the minimization stage consisted of 3000 steps of the steepest-descent algorithm followed by 3000 steps of the conjugate-gradient algorithm. During this process, a weak restraint of 10 kcal mol^−1^ Å^−2^ was applied to the backbone atoms of the proteins and the atoms of the drug. A second round of minimization was subsequently performed, during which all restraints on the solute were removed. Molecular-dynamics (MD) simulations were conducted in several stages. Initially, the system was subjected to a restrained MD simulation in the canonical ensemble (NVT) for 10 ps, during which the temperature was increased to 150 K. Restraints were applied to the protein backbone atoms and the drug atoms. The system was then further heated to a production temperature of 300 K over 50 ps, transitioning to the isothermal–isobaric ensemble (NPT) with a pressure of 1 atm regulated by the Berendsen barostat. To gradually relax the system, five additional short MD simulations of 100 ps each were performed, during which the restraints were progressively reduced by 2 kcal mol^−1^ Å^−2^ per step. Once all restraints had been removed, a 10 ns MD simulation was conducted under NPT conditions to ensure full equilibration of the system. Subsequent production runs were performed with pressure maintained using the Monte Carlo barostat. Electrostatic interactions throughout the simulations were calculated using the particle mesh Ewald (PME) algorithm. The temperature was regulated via the Langevin thermostat, with a collision frequency of 2.5 ps^−1^. The *SHAKE* algorithm was applied to constrain bond lengths involving H atoms, and additionally the hydrogen mass repartitioning scheme (Hopkins *et al.*, 2015 ▸) was followed, allowing the use of a 4 fs integration time step during the final equilibration and production stages. During the heating stage, a shorter integration time step of 1 fs was employed.

All analyses were performed using the *cpptraj* program of *AMBER* 2024 and in-house-developed Python scripts. To visualize the trajectories and generate the images, the *UCSF Chimera* (Pettersen *et al.*, 2004 ▸) and *VMD* (Humphrey *et al.*, 1996 ▸) software were employed. All unrestrained MD and raMD simulations (detailed below) were performed using the *pmemd* program of *AMBER* 2024, while the enhanced sampling simulations were performed with *GROMACS* 2022.5 (Abraham *et al.*, 2015 ▸) patched with the open-source, community-developed PLUMED library (version 2.9; Bonomi *et al.*, 2019 ▸; Tribello *et al.*, 2014 ▸). Simulations were executed on the Leonardo supercomputer (CINECA, Bologna, Italy) as well as on an in-house (CPU/GPU) hybrid cluster.

### 3.5. Random-accelerated MD (raMD)

Random-accelerated molecular dynamics (raMD), as per the *AMBER* implementation, is a technique used to efficiently sample drug-dissociation pathways. It introduces a random, artificial force on the drug during MD simulations, at defined intervals, accelerating its movement along dissociation pathways that may be challenging to observe on standard MD timescales. Multiple (40) raMD simulations were performed, where the random force (ramdboost = 21.5 Å ps^−2^) was applied at 20 ps intervals. The simulations were stopped either when drug unbinding was observed (the distance from the drug center of mass and the center of mass of the binding site was greater than 30 Å) or after 60 ns. A second set of raMD simulations (20) with an increased random force (ramdboost = 25 Å ps^−2^) was performed only for the system with the drug in the crystal orientation, to see whether a larger force could facilitate the unbinding.

### 3.6. On-the-fly probability enhanced sampling with metadynamics-like target distribution

On-the-fly probability enhanced sampling (OPES) with a metadynamics-like (metaD) target distribution (Invernizzi & Parrinello, 2020 ▸) is a method used to enhance the sampling of rare events in molecular simulations, particularly when exploring high-dimensional free-energy landscapes. In this approach, a biasing potential is applied in real time based on the evolving distribution of collective variables (CVs) of interest. The target distribution is chosen to be similar to that used in metaD, aiming to flatten the free-energy surface along the CVs by biasing the system towards under-sampled regions. The bias is updated as the simulation progresses, and the system is driven to explore areas of phase space that would otherwise be difficult to sample in conventional simulations. The first CV selected to drive the phase exploration (CV1) was the distance between the center of mass (COM) of the drug and the COM of the residues forming the inner region of the binding cavity. The distance between the drug COM and the COM of residues placed at the end of the unbinding pathway identified from the raMD simulations was selected as the second CV. The biasing potential was updated every 2 ps, and the initial estimated barrier was set to 25 kcal mol^−1^. To enhance the phase-space exploration, 32 walkers (parallel simulations) were run, each contributing to accumulate 1.280 µs of aggregate data collection. To prevent the undesirable unbinding of the drug during the OPES–metaD simulations, an upper wall (KAPPA = 40 kcal mol^−1^) was imposed, restraining CV1 to be less than 20 Å.

### 3.7. Synthesis of compound UPAR-1109

UPAR-1109 was synthesized as previously reported (Onajole *et al.*, 2013 ▸). The analytical data (^1^H-NMR, ^1^^3^C-NMR and HRMS) are consistent with those previously reported in the literature.

## 4. Results

### 4.1. Crystal structure of *M. smegmatis* MmpL3 in complex with UPAR-1109

**Figure 3:**
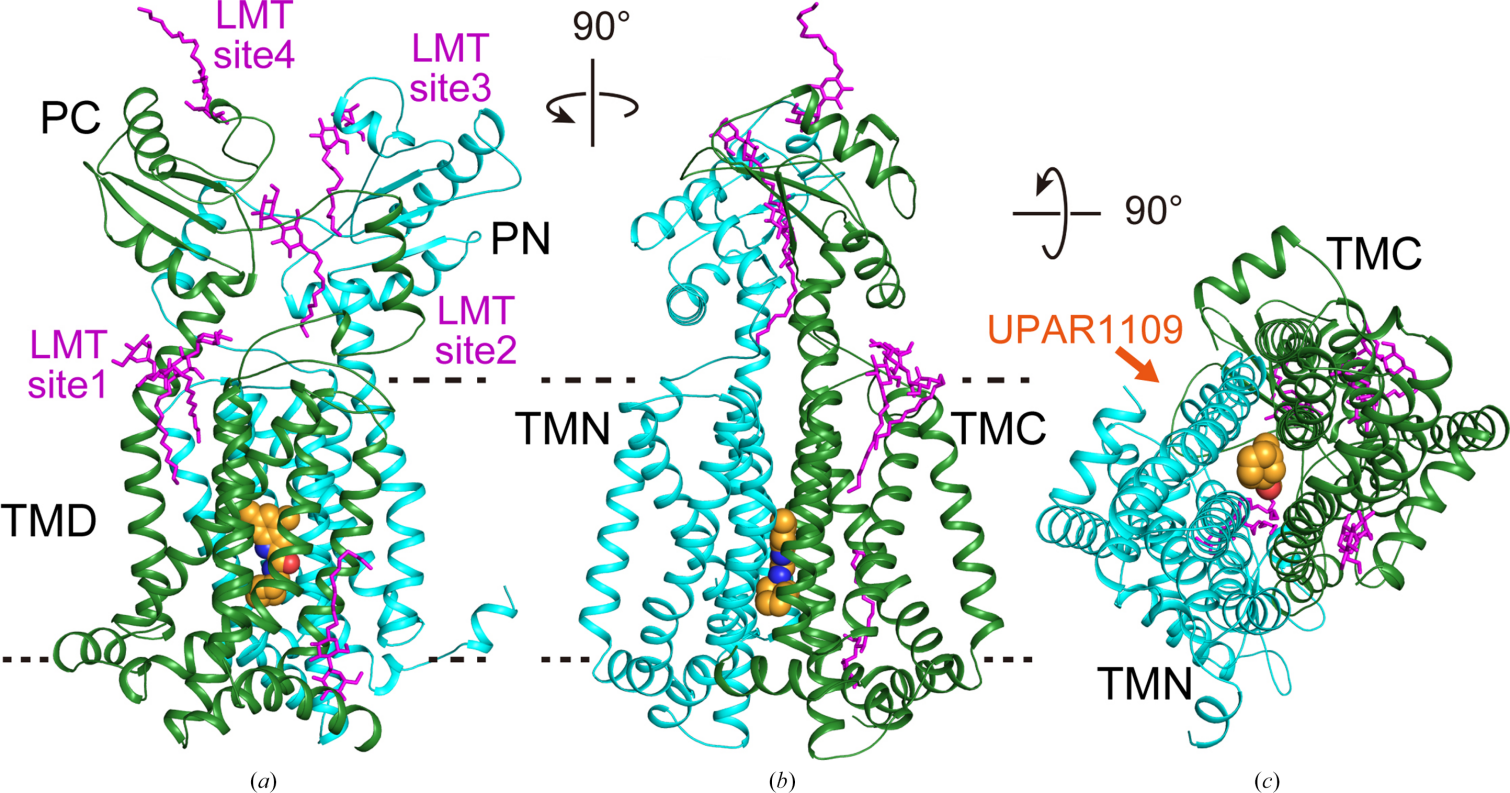
Overall structure of MmpL3 from *M. smegmatis* in complex with UPAR-1109. (*a*, *b*) Cartoon representation of MmpL3 viewed parallel to the membrane plane. (*c*) View from the cytosolic side. The N- and C-terminal halves are colored green and cyan, respectively. The UPAR-1109 molecule is shown in CPK representation with the positions of the C, O and N atoms shown in orange, red and blue, respectively. The LMT molecules are shown in stick representation and colored magenta.

To investigate the excellent antitubercular activity of UPAR-1109 for MmpL3 in detail, we crystallized MmpL3 (1–733) from *M. smegmatis* in complex with UPAR-1109 and determined its structure at 2.15 Å resolution (Fig. 3 ▸, Table 2 ▸). The overall structure was very similar to those reported previously (r.m.s.d. < 2 Å; Supplementary Fig. S1); however, the improved resolution allowed us to both build a more reliable model based on the electron-density map and to ensure greater accuracy in the subsequent computational approaches. Besides, this model also provided interesting structural insights into the functional aspects, such as the mechanism of inhibition by UPAR-1109 and the substrate-transport mechanism.

**Figure 4:**
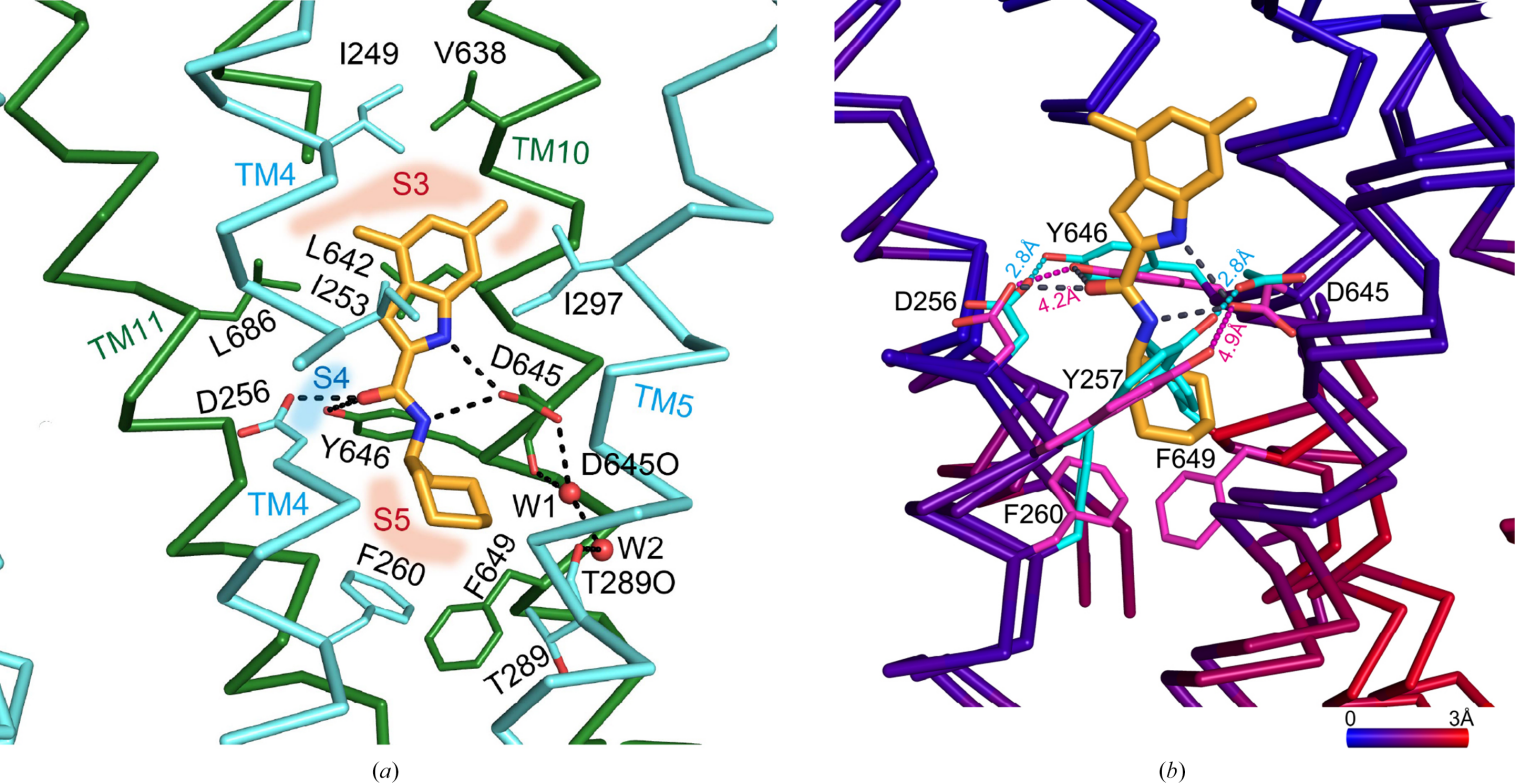
The UPAR-1109 binding site is shown in ribbon representation. (*a*) Close-up view of the UPAR-1109 binding site colored as in Fig. 3 ▸. The side chains that interact with UPAR-1109 are shown, and hydrogen bonds are shown as dotted lines. In addition, the water molecules that form part of the proton-translocation pathway are labelled W1 and W2. The zones where aliphatic and aromatic amino-acid residues that interact with UPAR-1109 via hydrophobic interactions are concentrated are indicated as S3 and S5, respectively. The polar interaction site involving Asp–Tyr is indicated as S4. (*b*) Overlay of the UPAR-1109-bound structure (PDB entry 9kbe) and the apo structure (PDB entry 6ajf). The Asp–Tyr pairs, important residues involved in proton translocation, as well as two aromatic residues (Phe260 and Phe649), are highlighted in magenta (PDB entry 9kbe) and cyan (PDB entry 6ajf). The distances between OD1 of Asp and OH of Tyr of each pair are also shown in Å. Ribbons are colored according to the superimposed C^α^ r.m.s.d. values of the corresponding locations. A scale bar showing the color gradient with the corresponding r.m.s.d. value is shown.

Like the compounds NITD-349 and ICA38, UPAR-1109 is bound to the cavity formed in the center of the pseudo-twofold symmetry formed by the N-terminal half (TMN) and C-terminal half (TMC) of TMD (Figs. 3 ▸*c* and 4 ▸*a*). As found in the NITD-349-bound structure, the distance between TMN and TMC increases by about 2 Å. The indole of UPAR-1109, methylated at positions R4 and R5, forms hydrophobic interactions with aliphatic amino acids such as Ile253, Ile429, Ile297, Leu642, Val638 and Leu686. In addition, the cycloheptane bound to R1 of the carboxamide on the opposite side of the compound intervenes in the T-shaped π-electron interaction between Phe260 and Phe649 and changes the side-chain rotamer of Phe649 (Fig. 4 ▸*b*). As a result, Phe260 also changes the position of its side chain and forms a hydrophobic interaction with the aromatic group; this leads to a change in the relative position of TM10 (Fig. 4 ▸*b*).

In addition, the N atoms of the indole ring and the O atom of the carboxamide of UPAR-1109 form hydrogen bonds to the carboxyl group of Asp645, the carboxyl group of Asp256 and the hydroxyl group of Tyr646. These aspartic acid and tyrosine residues have been shown in previous studies to be important in the proton-permeation pathway across the membrane (Zhang *et al.*, 2019 ▸; Bernut *et al.*, 2016 ▸). In the apo structure (PDB entry 6ajf), the carboxyl and hydroxyl groups of Asp256 and Tyr646 and of Asp645 and Tyr257 are both 2.8 Å apart (Fig. 4 ▸*b*, cyan dotted lines), suggesting the formation of a hydrogen bond, but UPAR-1109 binding fixes their distances at 4.2 and 4.9 Å, respectively (Fig. 4 ▸*b*, magenta dotted lines). We also observed a water molecule (Fig. 4 ▸*a*; W1) bound to the carboxyl group of Asp645. This water also binds to the carbonyl O atom of the main chain as well as to another water molecule (Fig. 4 ▸*a*; W2), which binds to the main-chain carbonyl O atom of Thr289 and almost reaches to the cytoplasmic side.

**Figure 5:**
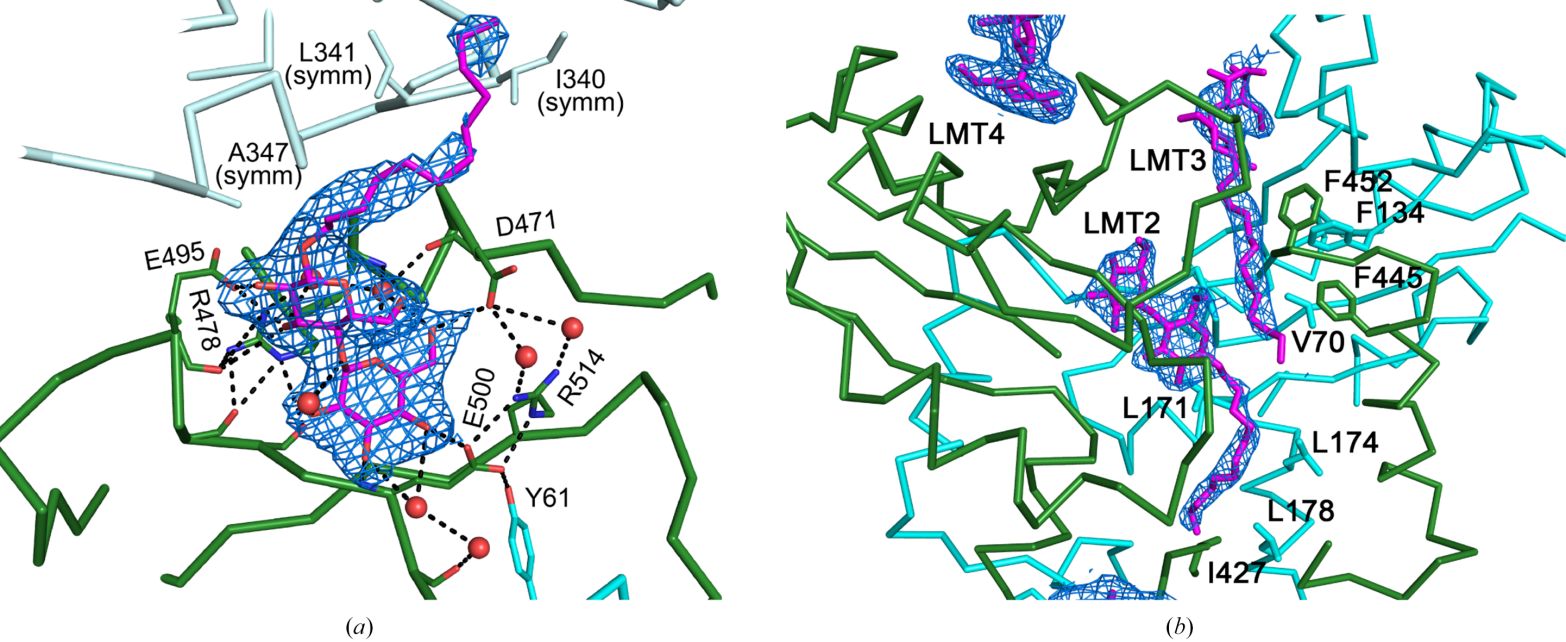
Stick models with electron-density maps of bound dodecyl maltoside (LMT) at the exit binding site (site 4) (*a*) and sites 2 and 3 (*b*). The coloring of the model is the same as in Fig. 3 ▸. An adjacent symmetry-related molecule in the crystal lattice is represented in a light color. Interacting residues and water molecules are represented by stick models and red spheres, respectively. The 2*F*_o_ − *F*_c_ electron-density map for LMT is shown as blue mesh and contoured at 1.0σ. Stereopairs and the 2*F*_o_ − *F*_c_ electron-density map for LMT at 0.65σ are shown in Supplementary Fig. S4.

**Figure 6:**
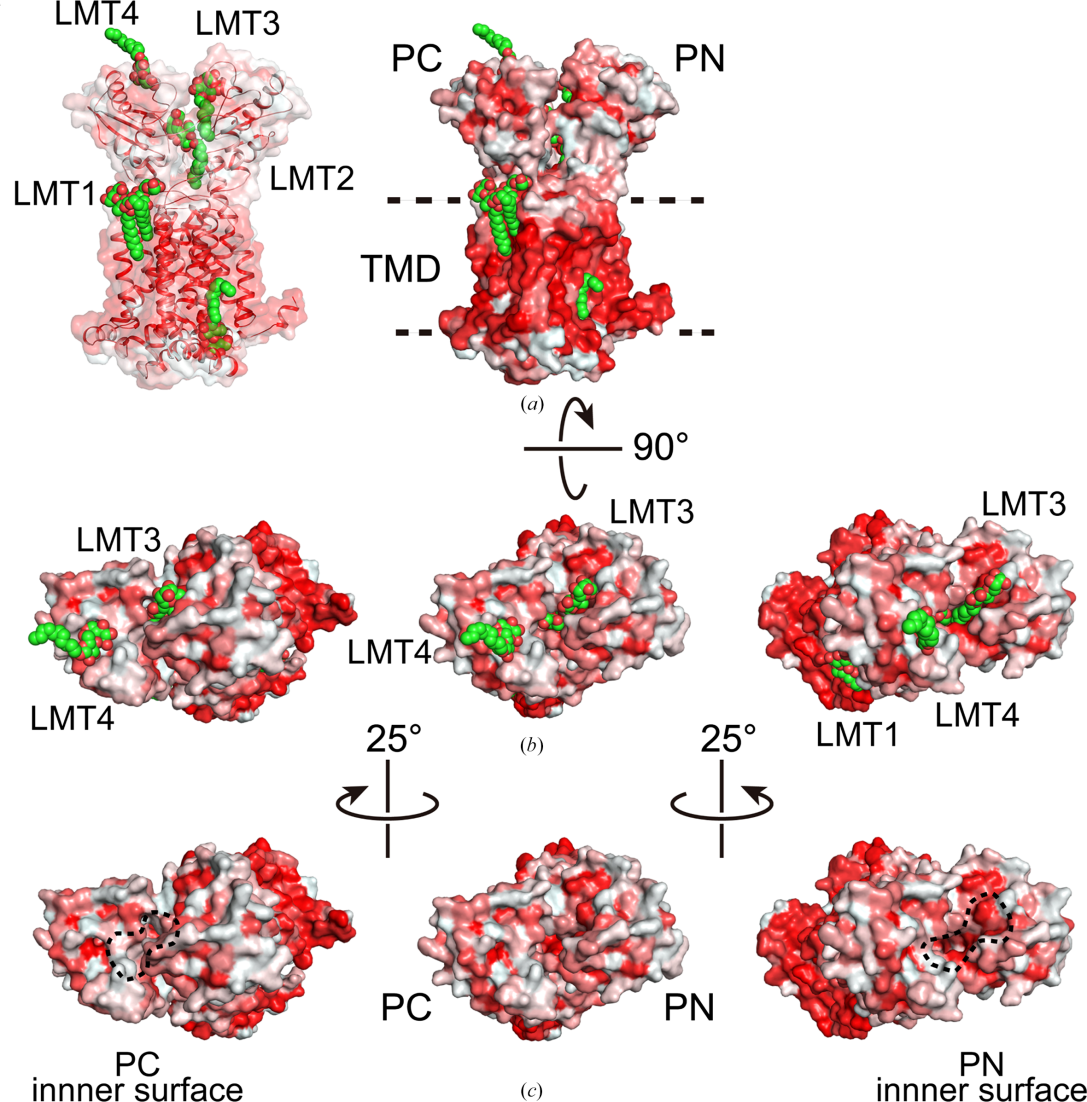
Representation of the hydrophobic surface. In this representation the red and white shades represent low and high hydrophobicity around the protein, respectively. Bound LMT molecules are shown in green CPK. (*a*) Side view (left: half transparent surface). (*b*) Top view: (left) view from 25° to the left, to make the inside of PD2 visible; (right) view from 25° to the right, to make the inside of PD1 visible. (*c*) To show the interior of the pocket clearly, the LMT model was eliminated and the inner surface of the pockets was enclosed with a dotted line.

**Figure 7:**
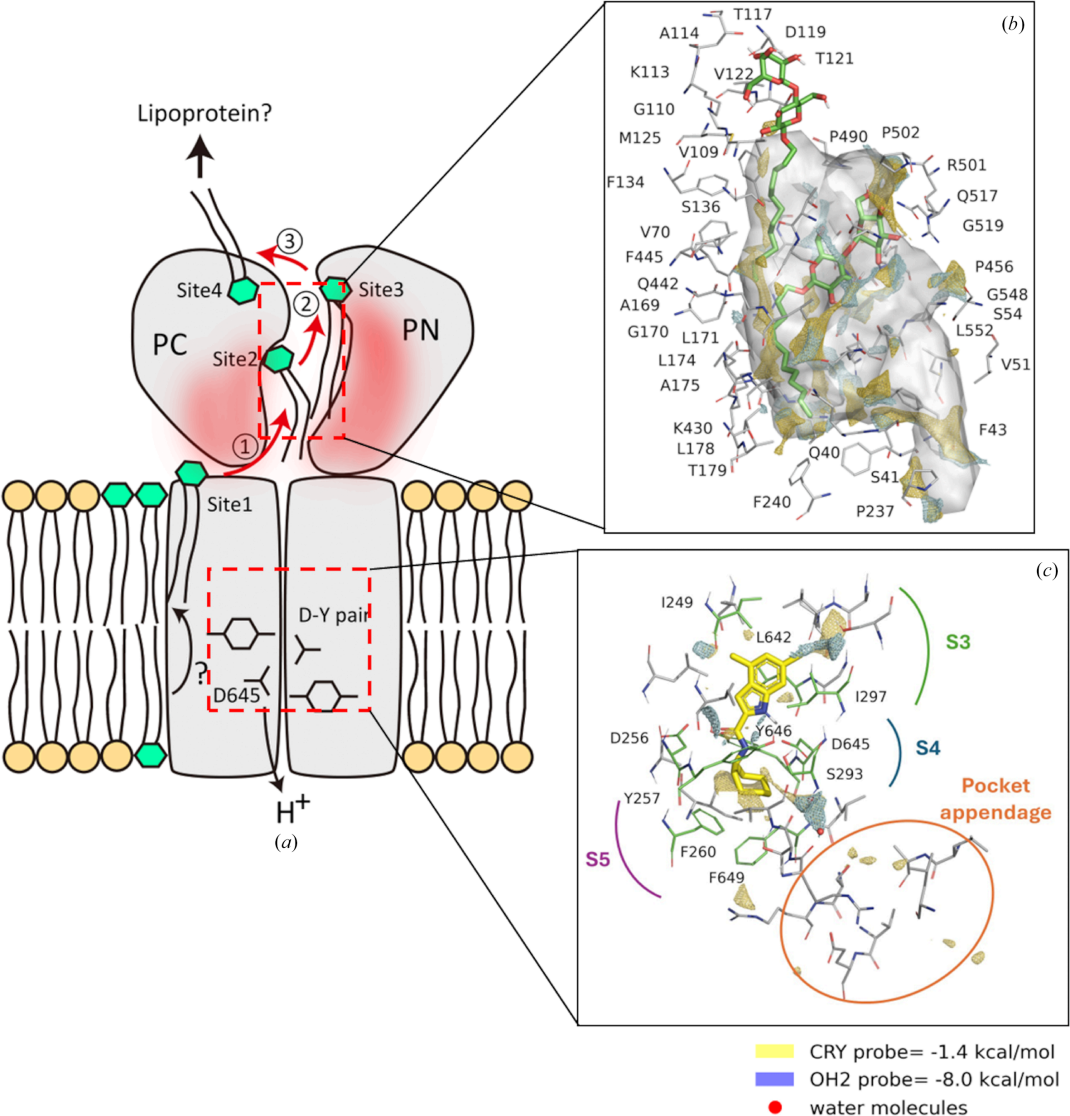
(*a*) Schematic illustration of the substrate-transport pathway by MmpL3 based on high-resolution structural analysis. The hydrophobic surface of the substrate-binding pocket formed between the two periplasmic domains (PN and PC) is shown in red. The important residues aspartic acid (D) and tyrosine (Y) in the TM domain, which are involved in proton translocation across the membrane, are depicted as a D–Y pair. Four binding sites are shown (named sites 1, 2, 3 and 4), based on the position of the LMT binding site observed in our crystal structure. The steps of transport, including the extraction of the substrate from the membrane (1) and its transport into the periplasmic binding pocket (2), and then the exit pathway with flip-flop (3), are numbered. (*b*) One of the largest binding sites is reported together with the *GRID* MIF: two units of LMT are partially aligned over hydrophilic (polar moiety) and hydrophobic (alkyl chain) MIFs, reported with cyan and yellow isocontour surfaces, obtained by using the probes OH2 (−8.0 kcal mol^−1^) and CRY (−1.4 kcal mol^−1^), respectively. (*c*) The main binding site of MmpL3, where UPAR-1109 binds, has both hydrophobic and polar hotspots; the hydrophobicity is mostly in subsites S3 and S5, whereas the polar hotspots are in subsite S4, where there is evidence of the good overlap between the water molecules (red) from the X-ray structure and the corresponding *GRID* MIF (probe OH2, reported in cyan).

The strong interaction can be explained by hydrophobic interactions on both sides of the indole moiety (S3 and S5 sites) and by the cycloheptane moiety of the carboxamide, together with polar interactions involving the Asp–Tyr residues at the S4 site (Fig. 4 ▸). The inhibition mechanism of UPAR-1109 involves direct interference with the proton-relay network located in the central transmembrane domain of MmpL3. By binding to this essential pocket, the inhibitor interacts with the conserved tyrosine residues (Williams & Abramovitch, 2023 ▸). This binding specifically disrupts the Asp–Tyr pairing, effectively breaking the hydrogen-bonding network required for the translocation of protons (H^+^) that powers the function of the transporter. Because these dyads act as a conformational electrostatic switch, their disruption prevents the proton motive force from supplying the energy necessary for substrate translocation, thereby arresting the transport of mycolic acids to the cell wall. We obtained interesting results not only in the TM region but also in the periplasmic region. In the published structures of MmpL3, the substrate (TMM), PE and detergents, which are substrate analogs, have been observed in the TMD and the periplasmic domain (Supplementary Fig. S1; Zhang *et al.*, 2019 ▸; Su *et al.*, 2019 ▸; Alsayed *et al.*, 2021 ▸). This suggests a proposed mechanism of substrate translocation from the outer leaflet of the membrane into the periplasmic domain (Alsayed *et al.*, 2021 ▸). Binding of detergent molecules was observed in the V-shaped valley formed by TM7, TM8 and TM9 (Fig. 3 ▸, site 1) and the periplasmic cavity formed between PN and PC (Fig. 3 ▸, sites 2 and 3). The acyl chains of the detergents bound to these sites (sites 1–3) extend towards the cytoplasmic side. In addition to these sites, we have also found a novel binding site on the proximal side of the periplasmic domain (Fig. 3 ▸, site 4). Surprisingly, the LMT bound to site 4 had its orientation flipped, with the acyl chain pointing outwards from the cell. There was a marked hydrophilic interaction between the maltose moiety of LMT and polar side chains (Asp471, Arg478, Glu495, Glu500 and Arg514) in PC directly and via bound water molecules (Fig. 5 ▸), but there was no marked hydrophobic interaction between the acyl-chain moiety and inner surface of PC, and the acyl chain was exposed on the outside of the molecule. When transported from site 1 to sites 2 and 3 through the gap between PC and the membrane, the acyl chain is pulled straight out from the outer layer of the cell membrane, and the acyl chain interacts with the hydrophobic surface of the pocket formed by PN and PC while remaining oriented in the same direction and facing the inside of the cell. When comparing the residues on the PN and PC surfaces in the periplasmic cavity, the PN surface is more hydrophobic, but some of the hydrophobic amino acids on the PC surface have been replaced with hydrophilic residues (Fig. 6 ▸). Therefore, when the hydrophilic part of the substrate pulled into the cavity is transferred from PN to PC, it is thought that the acyl chain will invert due to the lack of hydrophobic interactions with the PC surface (Fig. 7 ▸).

### 4.2. Characterization of the MmpL3 binding pocket in the proton flux-regulating region

The coordinate system described above (PDB entry 9kbe) was compared with the 3D structures of MmpL3 complexed with most of the currently known inhibitors: they all share the same periplasmic central cavity, but they differ in their engagement with the cavity volume. Initial visual inspection revealed substantial overlap between the identified pockets, with some notable differences. Specifically, PDB entries 6ajg, 6ajh, 6ajj and 7c2n display a regular, ‘oval-shaped’ pocket located within the transmembrane segment, whereas PDB entries 7wnx and 6aji exhibit a ‘cross-shaped’ pocket attributed to the presence of a ligand containing a diphenyl group. In contrast, PDB entry 9kbe displays a more complex pocket architecture characterized by an oval-shaped upper region and an appendage extending towards the cytosol. Analysis of the binding pocket across all available MmpL3 structures highlights its remarkable plasticity, as its shape varies depending on the bound ligand.

The geometry of ligand–target interactions in PDB entry 9kbe was further analyzed, with a focus on the key interactions of UPAR-1109, examined through virtual reality using the *Nanome* platform (https://docs.nanome.ai/nanome1x/plugins/docking.html); these interactions can be broadly categorized as polar and lipophilic. The polar interactions involve residues of the central hydrophilic subsite S4, where Asp and Tyr residues interact by forming hydrogen bonds to the indole and amide N atoms of UPAR-1109, consistent with previous findings for other inhibitors (Zhang *et al.*, 2019 ▸; Su *et al.*, 2021 ▸). Such polar contacts within the hydrophilic subsite are critical for MmpL3 inhibition (Zhang *et al.*, 2019 ▸; Su *et al.*, 2021 ▸; Alsayed *et al.*, 2021 ▸); disrupting the two hydrogen bonds formed between the two Asp–Tyr pairs impairs the ability of the protein to regulate proton flux across the membrane, a process essential for providing the energy required for mycolic acid translocation to the cell wall (Parish, 2022 ▸). Lipophilic interactions, in turn, involve the other two subsites, S3 and S5: subsite S3 is located in the upper portion of the pocket (as depicted in Fig. 7 ▸*c*), where hydrophobic interactions are established between the indole of UPAR-1109 and aliphatic residues such as Leu and Ile, in agreement with previous studies (Alsayed *et al.*, 2021 ▸). In contrast, the lower hydrophobic subsite S5 contains aromatic Phe residues that engage in hydrophobic interactions with the cycloheptane moiety of UPAR-1109 (Alsayed *et al.*, 2021 ▸).

**Figure 8:**
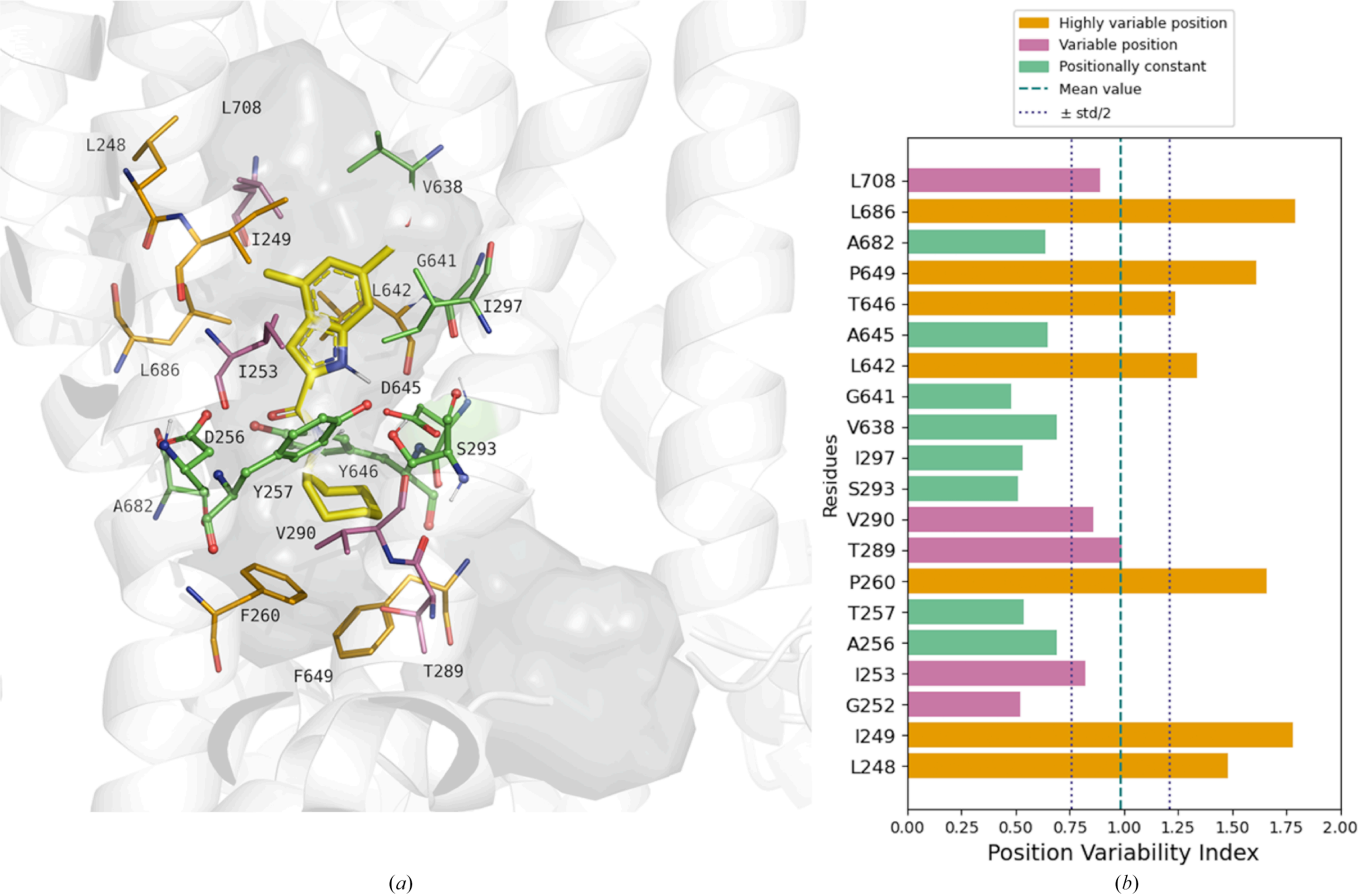
(*a*) Illustration of the binding pocket of MmpL3 in PDB entry 9kbe. The style of residues reflects the polar (ball-and-stick representation) or hydrophobic (stick representation) character of their lateral chain, whereas they are colored based on their positional variability: orange for residues with ‘highly variable position’, purple for residues with ‘moderately variable position’ and green for ‘positionally constant’ residues. (*b*) Bar plot for the positional variability index, obtained for 20 residues of the MmpL3 pocket, compared with thresholds.

To evaluate the pocket variability across crystal structures, we analyzed the position of amino-acid residues forming the binding pocket across different MmpL3 structures. Specifically, we compared the spatial coordinates of pocket residues in various crystal structures (PDB entries 7wnx, 7c2m, 7c2n, 6ajg, 6ajh, 6aji, 6ajj and 9kbe) to distinguish residues with significant positional shift from those maintaining a consistent position. Such calculations are based on averaged atomic Euclidean distance values, calculated through the atomic coordinates across all the pocket pairs; thus, for each residue, the resulting ‘position variability index’ reflects how much its position varies across the available crystallographic conformations. These indices were benchmarked against three threshold values corresponding to the mean and standard deviation across all the calculated values for all of the residues (mean + ½ s.d., mean and mean − ½ s.d.), allowing the classification of residues as expressing high/medium/low positional variability. This analysis revealed that the most variable residues are Leu and Ile in subsite S3, Tyr in the hydrophilic S4 subsite and Phe, Thr and Val in subsite S5 (Fig. 8 ▸).

### 4.3. Rationale for computational studies

Given the observed differences in the pockets of our MmpL3 structure (PDB entry 9kbe) compared with existing structures, we explored the binding modes of UPAR-1109 through computational studies. Using widely adopted docking software capable of accounting for both rigid and flexible receptor conformations, we predicted the binding modes of known inhibitors across the available MmpL3 structures: redocking experiments confirmed the robustness of the method, as the predicted poses closely reproduced the crystallographic orientations. To further elucidate the dynamic stability of different binding modes predicted by docking for UPAR-1109 in PDB entry 9kbe, we performed both unbiased and biased molecular-dynamics simulations.

### 4.4. Molecular interaction fields to detect hotspots in the MmpL3 binding pockets

MmpL3 binding pockets were characterized using *GRID* (Goodford, 1985 ▸; Tortorella *et al.*, 2021 ▸) molecular interaction fields (MIFs) to identify interaction hotspots; for this purpose, we used the following *GRID* probes: CRY for hydrophobicity and OH2 for water (able to accept and donate hydrogen bonds), as well as N1 and O as probes with hydrogen-bond donor and acceptor groups, respectively. The resulting MIFs were visually inspected and are shown as isocontour surfaces in Fig. 7 ▸(*c*) (and Supplementary Figs. S2 and S3).

X-ray data revealed two water molecules (W1 and W2) within the binding site of UPAR-1109; these are likely to be ‘structural waters’, as confirmed computationally using the OH2 probe: both lie within a well defined hydrophilic region, overlapping the corresponding MIF at very low energy levels. Consequently, these water molecules were retained in the subsequent docking studies. Using the same approach, we also analyzed the UPAR-1109 binding pocket to validate the hydrophobic and polar character of its subsites, consistent with prior literature.

Finally, we also examined an additional pocket in the upper part of the protein, using the CRY and OH2 probes to evaluate its hydrophilic and hydrophobic features. We observed that this pocket is characterized by highly hydrophilic regions (OH2 probe = −9.50) as well as by extended hydrophobic regions, as shown by the MIF of the CRY probe (Fig. 7 ▸*b*). Overall, the pocket extension, its irregular shape and amphiphilic character suggest its capacity to accommodate ligands bearing both polar and lipophilic moieties, highlighting its versatility in substrate binding. Thus, the presence of LMT, even in multiple orientations, is the consequence of the nature of this large pocket.

### 4.5. Docking studies

Each ligand was docked into its corresponding MmpL3 structure and the predicted binding poses were compared with the experimental poses using the r.m.s.d.; such values, for drug-like small molecules, are usually considered excellent when they are below 1 Å and acceptable around 2 Å, whereas larger r.m.s.d. values usually correspond to largely imprecise poses.

**Figure 9:**
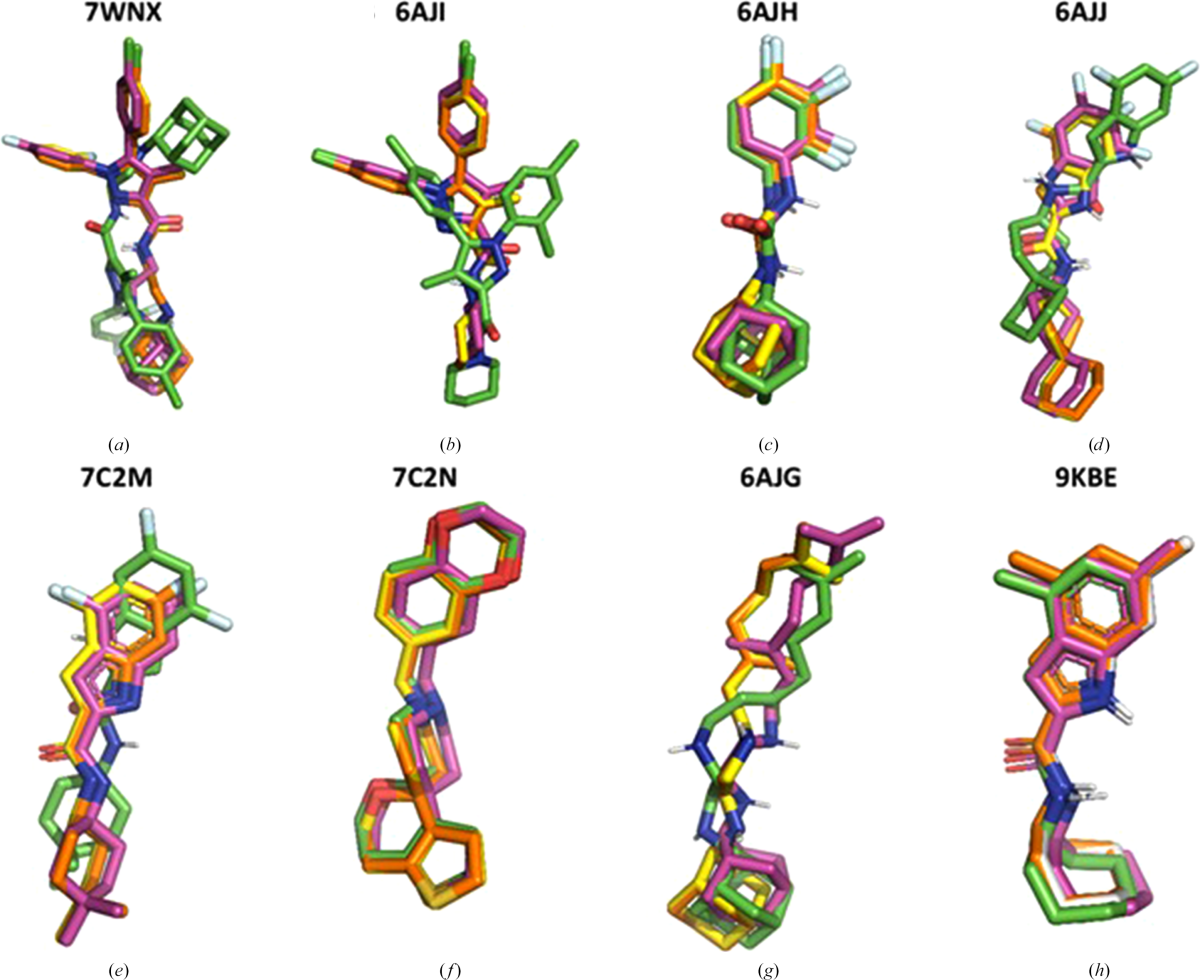
Results from redocking of eight MmpL3 inhibitors with three different methods. Color codes applied to C atoms are as follows: rsg, yellow; rlg, orange; fsg, green. The corresponding experimental poses are in pink. (*a*) Protein, PDB entry 7wnx; ligand, ST004. (*b*) Protein, PDB entry 6aji; ligand, rimonabant. (*c*) Protein, PDB entry 6ajh; ligand, AU1235. (*d*) Protein, PDB entry 6ajj; ligand, ICA38. (*e*) Protein, PDB entry 7c2m; ligand, FFU. (*f*) Protein, PDB entry 7c2n; ligand, SPIRO. (*g*), Protein, PDB entry 6ajg; ligand, SQ109. (*h*) Protein, PDB entry 9kbe; ligand, UPAR-1109.

Rigid docking using the ‘small grid’ (rsg) yielded excellent results across all MmpL3 structures, as shown by the close overlap between the first top-ranked predicted poses and the experimental orientations (r.m.s.d. values typically <1 Å, exceeding 2 Å only for SQ109). In contrast, flexible docking with the same ‘small grid’ (fsg) produced more variable results, since accurate poses were achieved only for three ligands (SPIRO, AU1235 and UPAR-1109), while the remaining ligands exhibited higher conformational freedom within the pocket, resulting in poorer agreement with crystallographic data. R.m.s.d. values exceeded the symbolic threshold of 2 Å for five of the eight ligands, with only SPIRO and UPAR-1109 achieving an r.m.s.d. below 1 Å, whereas those for AU1235 were between 1 and 2 Å. Finally, rigid docking with the ‘large grid’ (rlg) yielded results comparable to those from the smaller grid, indicating that across the available MmpL3 structures, the preferred inhibitor binding sites reside within the proton-flux regulating region. R.m.s.d. values are reported in Table 3 ▸, whereas Fig. 9 ▸ reports the docking poses with the three methods.

Notably, this analysis considers only the first top-ranked pose for each ligand–target pair; in the case of the PDB entry 9kbe UPAR-1109 complex, all three docking protocols identified the pose most consistent with the experimental structure as the best-ranked solution, a trend observed for most of the protein–ligand pairs. When extending this observation to other longitudinally shaped MmpL3 inhibitors (for example ICA38, AU1235 and SQ109), we noticed that the presence of two hydrophobic portions in these molecules allows, in principle, two possible orientations within the pocket. Interestingly, both the experimental data and the Vina scoring function consistently favor the orientation in which the aliphatic portion of the ligand faces the cytosol, while the aromatic portion points towards the inner transmembrane region.

Surprisingly, the aromatic and aliphatic characteristics of the key pocket residues appear to be reversed: the aliphatic moieties of the molecules (for example the spiro group of SPIRO, the adamantyl group of AU1235 and the cycloheptyl moiety of UPAR-1109) interact with the aromatic side chains of Phe260 and Phe649, whereas the aromatic moieties of the ligands lie near residues with aliphatic side chains, such as Ile249, Ile297 and Leu642.

This observation suggests the existence of two potential binding orientations for these linear inhibitors, though only one appears to be favored. Examination of alternative docking solutions (those with less relevant scores) revealed that the ‘reversed orientation’ of UPAR-1109 ranked fifth in rsg, seventh in rlg and third in fsg calculations. These findings prompted further computational analyses to clarify the energetic basis of this apparent inversion and to understand why only one binding mode is experimentally observed.

### 4.6. Molecular-dynamics studies

To further investigate the differences between the two binding modes of UPAR-1109, that is the crystallographic and the reversed orientation, MD simulations were performed with the drug positioned in both observed orientations within the binding site of MmpL3. To verify that the absence of positional restraints during the 500 ns production phase (uMD) did not compromise the global fold of MmpL3 in our aqueous model, we monitored the backbone r.m.s.d. relative to the crystallographic structure along the uMD trajectories for the different ligand orientations. The r.m.s.d. traces show that the transporter remains structurally stable over the simulated timescale and does not exhibit progressive unfolding or large-scale drift (Fig. 2 ▸). This provides an internal consistency check that the observed differences between binding orientations are not an artifact of gross structural destabilization in unrestrained simulations.

**Figure 10:**
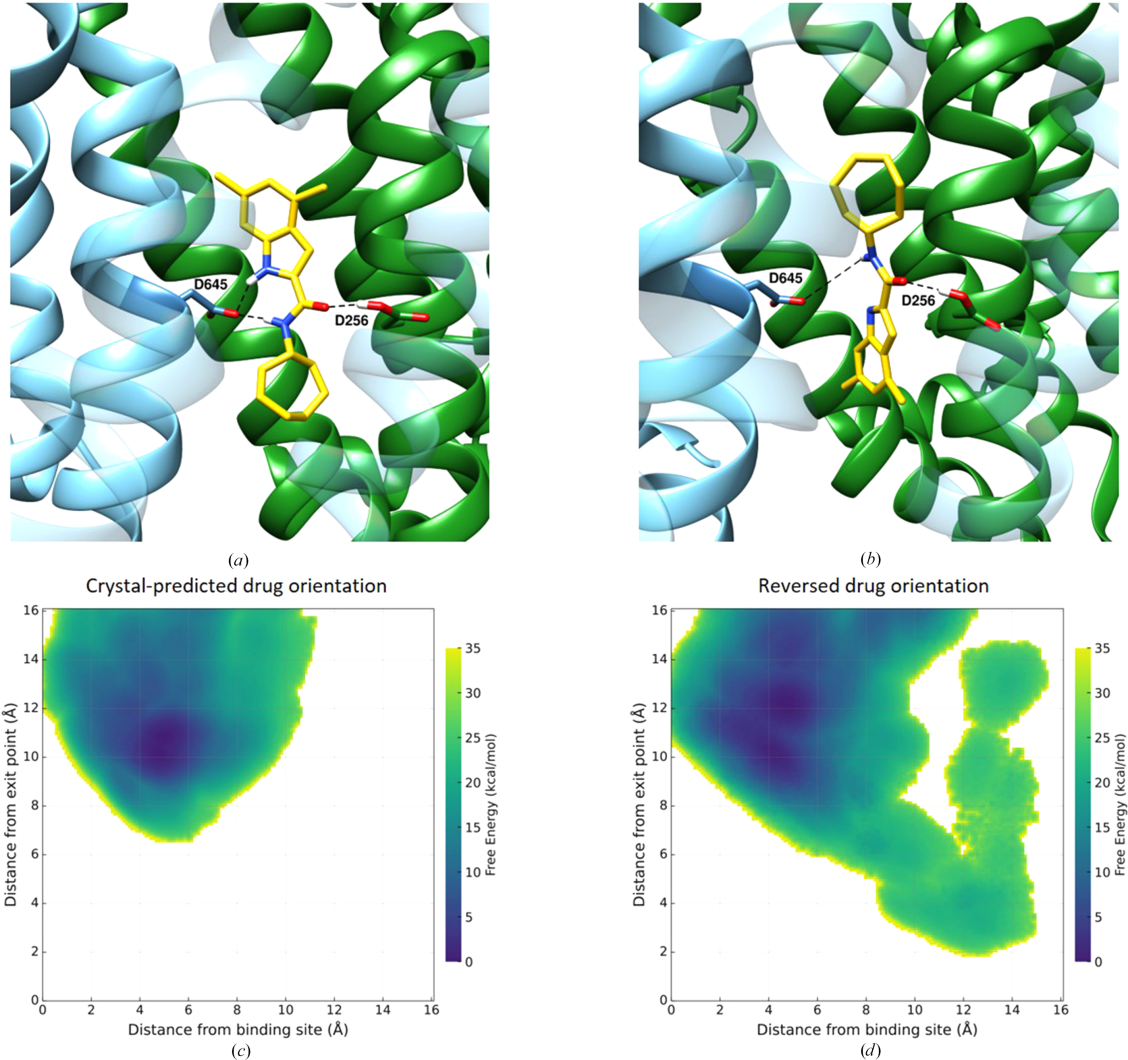
(*a*, *b*) Hydrogen-bond network stabilizing the drug during the uMD simulations. Protein and drug are depicted following the color scheme of Fig. 3 ▸, while hydrogen bonds are represented by dashed black lines. (*c*, *d*) Free-energy surfaces (FES) obtained from OPES–metaD simulations as a function of the distance between the drug and the binding site and the distance between the drug and the putative exit point.

**Figure 11:**
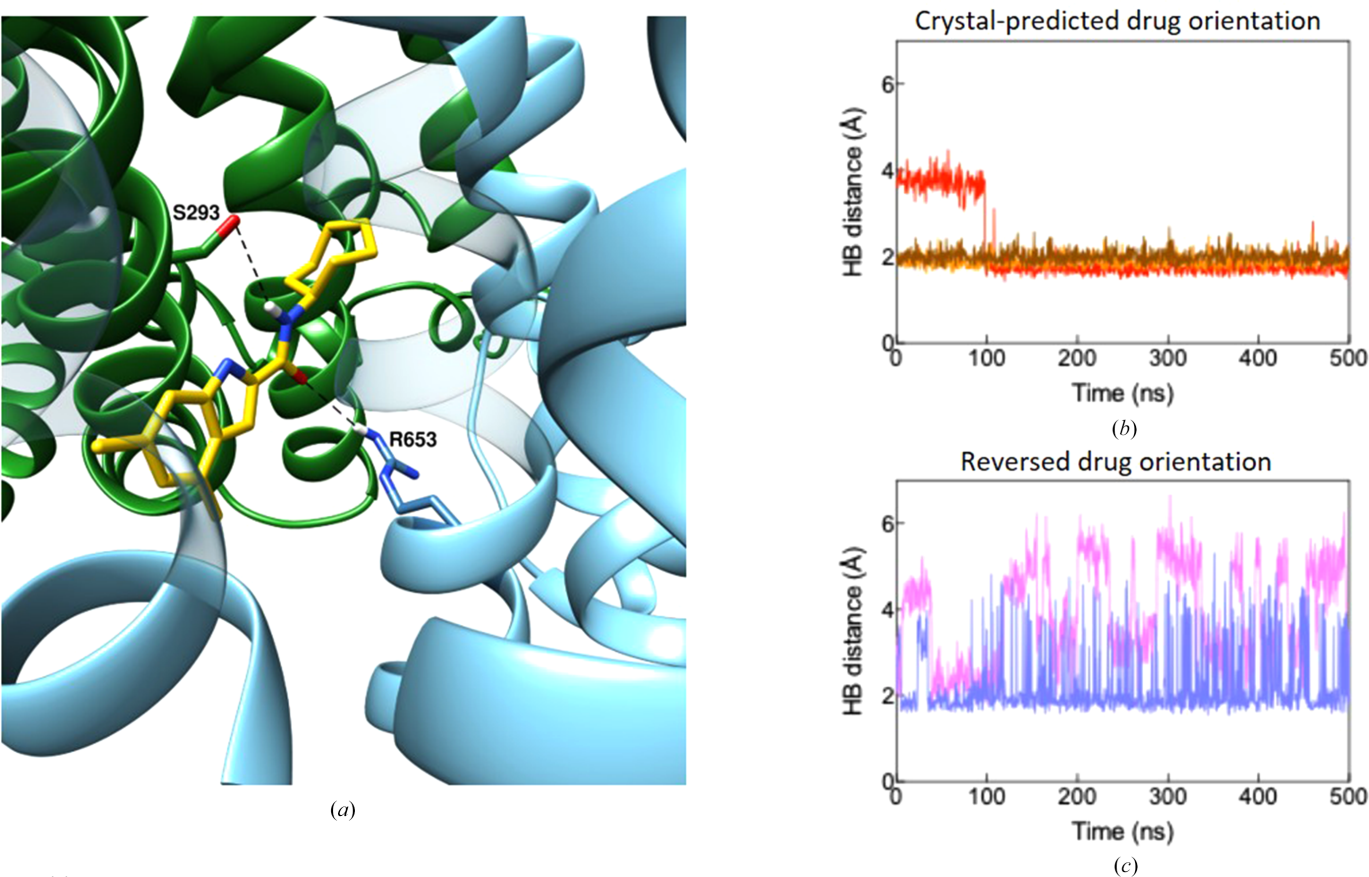
(*a*) Representative snapshot extracted during the unbinding of the drug from the reversed orientation in an raMD simulation. Two relevant hydrogen bonds responsible for the stabilization of the unbinding pathway are depicted with dotted black lines, while protein and drug are depicted following the color scheme of Fig. 3 ▸. (*b*, *c*) Distances between the atoms forming hydrogen bonds during uMD of the systems with the drug as in the crystal structure (*b*) and as predicted by the reversed orientation (*c*).

Such simulations indeed reveal significant differences in the dynamical stability of the drug–protein interactions. In the crystallographic orientation, where the aromatic group of UPAR-1109 is positioned towards the interior of the binding site, a robust hydrogen-bond network comprising three bonds was observed (Fig. 10 ▸*a*). These interactions remained highly stable throughout the 500 ns simulation (see Fig. 11 ▸*b*), indicating a strong and persistent interaction pattern. Conversely, in the alternate orientation, where the aromatic group of UPAR-1109 is directed outwards, only two hydrogen bonds formed (Fig. 10 ▸*b*). These bonds were markedly unstable, with frequent disruption and reformation during the simulation (see Fig. 11 ▸*c*). This instability suggests that the outward-facing orientation may not support the same level of structural complementarity or interaction strength as the crystallo­graphic orientation. Moreover, the presence of aromatic residues near the exterior of the binding site, which might have favored the docked pose, appears to be insufficient to compensate for the reduced hydrogen-bonding stability. These findings highlight the critical role of hydrogen-bond networks in stabilizing the UPAR-1109–MmpL3 complex.

To further investigate whether the higher stability observed for the crystallographic orientation of UPAR-1109 translates into a greater residence time within the MmpL3 binding site, random-accelerated molecular-dynamics (raMD) simulations were performed. This method applies a biasing force to enhance the sampling of rare events, such as ligand unbinding. This enables the estimation of binding stability on shorter timescales compared with those achievable with unbiased simulations. A total of 40 raMD simulations were conducted for each ligand orientation using an acceleration of 21.5 Å ps^−2^. In the crystallographic orientation, unbinding events were observed in only 12% of the simulations within 40 ns, suggesting a high binding stability. In contrast, the reversed orientation exhibited a significantly higher unbinding frequency, with 76% of simulations showing ligand escape under the same conditions. To assess whether the resistance to unbinding in the crystallographic orientation could be overcome by increased acceleration, an additional 20 raMD simulations were performed with a higher acceleration of 25.0 Å ps^−2^. Remarkably, the unbinding percentage remained unchanged, reinforcing the conclusion that the crystallo­graphic orientation forms a highly stable complex with MmpL3. These results further validate the superior stability of the hydrogen-bond network, and the overall binding environment, of the crystallographic orientation compared with the reversed orientation. This stability likely contributes to the reduced propensity for unbinding and highlights the importance of structural complementarity in achieving effective binding.

Interestingly, analysis of the raMD simulations in which unbinding occurred revealed a preferred exit pathway for UPAR-1109, particularly when oriented as in the docked pose. Along this pathway, transient hydrogen bonds were observed between UPAR-1109 and residues Arg653 and Ser293. These interactions may guide the ligand during its exit and potentially stabilize intermediate states along the unbinding trajectory. The presence of these interactions suggests that even during accelerated-simulation unbinding, the ligand retains specific interactions with the protein (see Fig. 11 ▸*a*).

To further elucidate the differences between the two orientations of UPAR-1109 in the MmpL3 binding site, simulations based on on-the-fly probability enhanced sampling (OPES) with a metadynamics-like (metaD) target distribution were performed. This advanced sampling method enhances the exploration of the free-energy landscape by applying a bias to the system in real time, guiding it towards a predefined probability distribution. OPES–metaD is particularly effective for identifying and characterizing energetically favorable states and transition pathways, making it ideal for studying binding and unbinding processes. Two collective variables (CVs) were selected for this study: the distance between the center of mass (COM) of UPAR-1109 and the COM of the most internal portion of the binding site, and the distance between the COM of UPAR-1109 and the COM of residues located in the outer region of the unbinding pathway identified in the raMD simulations. The free-energy surfaces (FESs) generated along these CVs reveal notable differences between the two configurations. For the crystallographic orientation of UPAR-1109 (Fig. 10 ▸*c*), the ligand primarily explores a region near the binding site, remaining confined and unable to exit even under the influence of the applied bias. The distances measured along the CVs remain consistent with the crystallographic pose, indicating strong and stable binding. In contrast, for the reversed orientation, the FES displays the existence of a second energy minimum at higher free-energy levels (Fig. 10 ▸*d*). This minimum is characterized by a greater distance from the internal region of the binding site and a reduced distance to the external residues identified in the unbinding pathway. Notably, this secondary minimum corresponds to a position where UPAR-1109 establishes hydrogen bonds to residues Arg653 and Ser293, as observed in the raMD simulations. These interactions stabilize the ligand along its unbinding pathway, further facilitating its escape from the binding site.

Combining the insights from raMD and OPES–metaD simulations, we propose that the reversed orientation of UPAR-1109 not only forms less stable interactions within the binding site, but it is also inclined to exit the binding site more easily. This results in a markedly reduced residence time for UPAR-1109 in this orientation, reinforcing the conclusion that the crystallographic pose is the more stable and biologically relevant configuration.

## 5. Discussion and conclusions

This study sheds light on the stability of the interactions of a potent indolecarboxamide, UPAR-1109, within the binding site of MmpL3, in order to gain useful structural information for the design of novel inhibitors; in fact, 80% of the resistance mutations lie inside or close to the inhibitor-binding pocket and studies with purified mutant proteins of MmpL3 showed a decrease in the binding or its abolition (Zhang *et al.*, 2019 ▸).

The comparative analysis of the transmembrane pockets across different MmpL3 structures revealed that while some residues maintain consistent positions regardless of the bound ligand, others exhibit significant variability to accommodate ligands of different sizes. In other words, most of the six C-terminal transmembrane helices undergo noticeable conformational changes upon inhibitor binding, and these changes disrupt the interactions of the two Asp–Tyr pairs critical for proton translocation, effectively blocking the proton motive force (PMF) required for substrate translocation.

The binding of all of the MmpL3 inhibitors known to date involves extensive hydrophobic interactions, which help to stabilize the position of the inhibitor within the cavity, and contribute to the inhibitory effects on MmpL3 function. When applied to the MmpL3–UPAR-1109 complex (PDB entry 9kbe), docking successfully reproduced the experimental pose, indicating that the pocket appendage does not influence the binding of UPAR-1109. However, guided by the study of hydrophobic interactions, we extended the analysis of two of the most relevant binding modes observed of UPAR-1109: using molecular-dynamics studies, we verified that the orientation observed experimentally is the most stable.

MmpL3 is a transporter for TMM but is also considered a type of flippase (Xu *et al.*, 2017 ▸). This refers to the activity by which substrates biosynthesized in the cytoplasm are transported across the membrane and further passed across the periplasm to the systems involved in mycomembrane biosynthesis, such as Ag85 (Belardinelli *et al.*, 2019 ▸). The role of the transmembrane domain, which shares a high-level structural motif with other RND transporter families such as HAE1/RND transporters, is clearly to convert the proton motive force across the membrane into molecular movement, which has been extensively studied in HAE1/RND transporters and also in HAE2, including MmpL3, and SecDF, suggesting the existence of a common mechanism (Xu *et al.*, 2017 ▸). The conformational changes in MmpL3 result in substrate uptake from the outer leaflet of the membrane into the substrate-translocation pathway (or pocket) formed between PN and PC and substrate export to the distal side of the molecule (Su *et al.*, 2021 ▸).

The substrate is taken up into the pocket between PN and PC through the substrate entrance formed between Ser423 in the TM loop and Asn524 in the PC domain (Su *et al.*, 2021 ▸). The inner surface of the pocket is rich in aromatic amino acids such as phenylalanine residues and aliphatic amino acids such as leucine and isoleucine, making it highly hydrophobic (Fig. 5 ▸*b*), and stabilizes the lipophilic substrate. The molecular motion induced by proton translocation through the TM domain propagates to the periplasmic domain. This, coupled with the rotational rigid-body motion of PN and PC, causes structural changes in the pocket. This might be the mechanism of substrate translocation from site 2 to site 3. Interestingly, in the TMM- and PE-bound structures, these substrates are already flipped within the pocket (Su *et al.*, 2019 ▸, 2021 ▸). LMT4, identified in this study, was observed in the flipped orientation (Fig. 5 ▸*a*). Additionally, there might be a slight difference in hydrophobicity between the inner surfaces of PN and PC (Fig. 6 ▸).

In comparing the hydrophobicity of the upper part of the pocket, the surface formed by Val70, Phe134, Phe445 and Phe452 exhibits higher hydrophobicity than the opposite surface along the intramolecular twofold-symmetry axis (Figs. 5 ▸*b* and 6 ▸). Consequently, at site 3, the acyl chain of LMT binds stably to these residues. However, when LMT crosses over to site 4, the pocket there contains fewer hydrophobic residues and is more hydrophilic. Consequently, at site 4, located in the upper part of the pocket, the acyl chain is not oriented towards the interior of the pocket. It is therefore speculated that this may result in flipping (Figs. 6 ▸ and 7 ▸). This could potentially explain the substrate-inversion mechanism. The mechanism based on the hydrophobicity difference might also apply to the flipping of TMM and PE within the periplasmic pocket. However, the binding mode and interaction of MmpL3 with lipoproteins that pass TMM at the distal end of the pathway in the MmpL3 also remains a question.

MmpL3 is an essential protein for mycobacterial physiology and pathogenesis, playing a critical role in cell-envelope biogenesis. It functions as a transporter for trehalose monomycolates, which are precursors in mycolic acid biosynthesis, a process vital for maintaining the integrity of the bacterial cell envelope. Depletion of MmpL3 disrupts this pathway, leading to a reduction in lipid availability, arresting cell division and ultimately causing rapid bacterial death. The sequence of MmpL3 is highly conserved across mycobacteria (Varela *et al.*, 2012 ▸), including the residues involved in binding, implying that MmpL3 inhibitors may have a broad spectrum of activity against multiple species (Varela *et al.*, 2012 ▸; Li, Schurig-Briccio *et al.*, 2014 ▸); an example of a nontuberculous mycobacterium (NTM) is *M. abscessus*, for which treatment options are severely limited.

Using a multidisciplinary approach, we studied the binding of UPAR-1109, an indolecarboxamide inhibitor of MmpL3: from X-ray crystallography, we observed significant differences between our co-crystallized structure and those reported in the existing literature; this prompted further investigation into potential entry pathways for the ligand within the binding site, carried out through computational simulations, including docking and molecular dynamics. We highlighted that UPAR-1109 binds very strongly by direct polar interaction with the Asp–Tyr pair, which is essential for proton transmission, in combination with both aliphatic and aromatic hydrophobic interactions.

To further assess whether the reversed orientation,suggested by docking and plausible from a chemical standpoint, could represent a viable binding mode, we conducted an extensive computational investigation combining classical and enhanced sampling molecular dynamics. Although the reversed pose is sterically accessible and initially forms plausible interactions, our simulations reveal that it lacks the stabilizing hydrogen-bond network observed in the crystallo­graphic orientation and shows a markedly higher tendency to dissociate. This is reflected both in unbiased simulations and in the significantly increased unbinding rates under accelerated conditions. Free-energy surface analyses confirm that the reversed pose populates higher energy metastable states along an exit pathway, stabilized only transiently by peripheral interactions. These findings demonstrate that the crystallo­graphic pose corresponds not only to a structurally compatible orientation but also to the thermodynamically most stable binding mode, thereby reinforcing its biological relevance.

Furthermore, the binding site is located between TM4 and TM10, the key site of RND molecular motion, *i.e.* on the pseudo-twofold-symmetry axis, and the tight binding to this site may have greatly reduced molecular fluctuations. The suppression of fluctuations by the binding of UPAR-1109 may have led to the tight packing of the crystal lattice and the significant improvement in resolution in this structural analysis.

Given the higher resolution and the presence of several LMT molecules binding to MmpL3, we could also speculate about the diverse roles of PC and PN of MmpL3, with their different distributions of hydrophobic residues, with consequences for a hypothetical mechanism to flip substrates in the periplasmic pocket.

Overall, this work not only presents the MmpL3 crystal with the best resolution so far, but also could deepen our understanding of the interactions of MmpL3 with inhibitors; this information might contribute to the development of more effective antituberculosis therapies, but the scope of this investigation may also extend to other mycobacterial strains, such as nontuberculous mycobacteria, and potentially to other bacterial species, given the similarity of these transport systems to those found in different bacteria.

## Supplementary Material

Supplementary Figures and Table. DOI: 10.1107/S2059798326003050/qe5012sup1.pdf

PDB reference: *M. smegmatis* MmpL3 in complex with UPAR-1109, 9kbe

## Figures and Tables

**Table 1:** PDB entries currently available in the Protein Data Bank archive, sorted by release date The first column reports whether crystals are in the apo form (—) or complexed with small molecules (lipids, glycolipids or inhibitors).

Ligand code (name)	Bacteria	PDB entry†	Extended PDB ID	Method‡	Resolution (Å)	Reference
L6T§	*M. smegmatis* mc^2^ 155	**6ajf**	pdb_00006ajf	X-ray	2.70	Zhang *et al.* (2019 ▸)
LMT§, 3RX (SQ109)¶	*M. smegmatis* mc^2^ 155	**6ajg**	pdb_00006ajg	X-ray	2.60	Zhang *et al.* (2019 ▸)
L6T§, 9ZF (AU1235)¶	*M. smegmatis* mc^2^ 155	**6ajh**	pdb_00006ajh	X-ray	2.82	Zhang *et al.* (2019 ▸)
L6T§, AY6 (rimonabant)¶	*M. smegmatis* mc^2^ 155	**6aji**	pdb_00006aji	X-ray	2.90	Zhang *et al.* (2019 ▸)
L6T§, J9E (ICA38)¶	*M. smegmatis* mc^2^ 155	**6ajj**	pdb_00006ajj	X-ray	2.79	Zhang *et al.* (2019 ▸)
-	*M. smegmatis* mc^2^ 155	**6n40**	pdb_00006n40	X-ray	3.31	Su *et al.* (2019 ▸)
LMT§, L9Q (phosphatidylethanolamine)††	*M. smegmatis* mc^2^ 155	**6or2**	pdb_00006or2	X-ray	2.59	Su *et al.* (2019 ▸)
L6T§, FFU (NITD-349)¶	*M. smegmatis* mc^2^ 155	**7c2m**	pdb_00007c2m	X-ray	3.10	Xu *et al.* (2017 ▸)
L6T§, FG0 (SPIRO)¶	*M. smegmatis* mc^2^ 155	**7c2n**	pdb_00007c2n	X-ray	2.82	Xu *et al.* (2017 ▸)
AV0 (LMNG)§	*M. tuberculosis*	**7nvh**	pdb_00007nvh	Cryo-EM	3.00	Adams *et al.* (2021 ▸)
0HJ (TMM)††	*M. smegmatis* mc^2^ 155	**7n6b**	pdb_00007n6b	Cryo-EM	2.66	Su *et al.* (2021 ▸)
T6D††	*M. smegmatis* mc^2^ 155	**7k7m**	pdb_00007k7m	X-ray	3.33	Su *et al.* (2021 ▸)
—	*M. smegmatis* mc^2^ 155	**7k8a**	pdb_00007k8a	Cryo-EM	3.65	Su *et al.* (2021 ▸)
—	*M. smegmatis* mc^2^ 155	**7k8b**	pdb_00007k8b	Cryo-EM	2.94	Su *et al.* (2021 ▸)
—	*M. smegmatis* mc^2^ 155	**7k8c**	pdb_00007k8c	Cryo-EM	4.27	Su *et al.* (2021 ▸)
—	*M. smegmatis* mc^2^ 155	**7k8d**	pdb_00007k8d	Cryo-EM	4.33	Su *et al.* (2021 ▸)
1I2 (ST004)¶	*M. smegmatis* mc^2^ 155	**7wnx**	pdb_00007wnx	Cryo-EM	3.36	Hu *et al.* (2022 ▸
—	*M. smegmatis* mc^2^ 155	**8qkk**	pdb_00008qkk	Cryo-EM	3.23	Couston *et al.* (2023 ▸)
LMT§, UPAR1109¶	*M. smegmatis* mc^2^ 155	**9kbe**	pdb_00009kbe	X-ray	2.15	This study

† Entries reported in bold were used in the analysis.

‡ Crystallography (X-ray) or cryo-electron microscopy (Cryo-EM).

§ Detergent (LMT, dodecyl-β-D-maltoside; L6T, lauryl-6-trehaloside; LMNG, lauryl maltose neopentyl glycol, 6-decanoyl trehalose).

¶ Inhibitor.

†† Lipid.

**Table 2:** Data-processing and refinement statistics for *M. smegmatis* MmpL3 in complex with UPAR-1109 (PDB entry 9kbe) Values in parentheses are for the highest resolution shell.

Data collection	
Beamline	BL44XU, SPring-8, Japan
Space group	*P*2_1_
*a*, *b*, *c* (Å)	84.29, 105.31, 89.47
α, β, γ (°)	90, 114.521, 90
Wavelength (Å)	0.900
Resolution (Å)	46.94–2.15 (2.18–2.15)
No. of reflections	
Observed	2011691
Unique	77682
Multiplicity	25.90 (7.07)
*R*_merge_	0.126 (>1.0)
CC_1/2_	0.996 (0.744)
Completeness (%)	99.91 (98.59)
〈*I*/σ(*I*)〉	12.4 (1.2)
Refinement	
Resolution (Å)	43.42–2.15
*R*/*R*_free_	0.2158/0.2424
R.m.s. deviation from ideal	
Bond lengths (Å)	0.003
Bond angles (°)	0.568
Average *B* factor (Å)	82.2
Ramachandran plot (%)	
Favored	98.89
Allowed	1.11
Disallowed	0.00

**Table 3:** Redocking results for the eight MmpL3 inhibitors in the corresponding target, and r.m.s.d. values for the best poses obtained by using three different docking settings: rsg, rigid and small grid; rlg, rigid and large grid; fsg, flexible and small grid

MmpL3 inhibitor	PDB code	R.m.s.d. (Å)		
		Docking rsg	Docking rlg	Docking fsg
ST004	**7wnx**	1.131	1.140	10.416
Rimonabant	**6aji**	0.833	0.842	5.475
AU1235	**6ajh**	1.200	1.340	1.508
ICA38	**6ajj**	0.895	0.907	2.564
FFU	**7c2m**	0.238	0.231	2.161
SPIRO	**7c2n**	0.858	0.835	0.812
SQ109	**6ajg**	2.255	2.284	2.044
UPAR-1109	**9kbe**	0.683	0.720	0.694

## Data Availability

Coordinates and structure factors for *M. smegmatis* MmpL3 with UPAR-1109 have been deposited in the Protein Data Bank with accession code 9kbe.
