# Supplementary material for: Gaining insights into MmpL3: combining structural and computational approaches to unlock transport and inhibitor-binding mechanisms
Source: Acta Crystallogr D Struct Biol. 2026 Apr 29;82(Pt 5):550–70. doi: 10.1107/S2059798326003050 (PMC13134001; doi:10.1107/S2059798326003050)
Supplement: Supplementary file 1 [file d-82-00550-sup1.pdf]

STRUCTURAL
BIOLOGY

Volume 82 (2026)

Supporting information for article:

Gaining insights into MmpL3: combining structural and computational approaches to unlock transport and inhibitor-binding mechanisms

Satoshi Murakami, Domenico Marson, Eiki Yamashita, Bruno Broshka, Ui Okada, Maho Aoki, Giannamaria Annunziato, Erik Laurini, Emanuele Carosati and Marco Pieroni

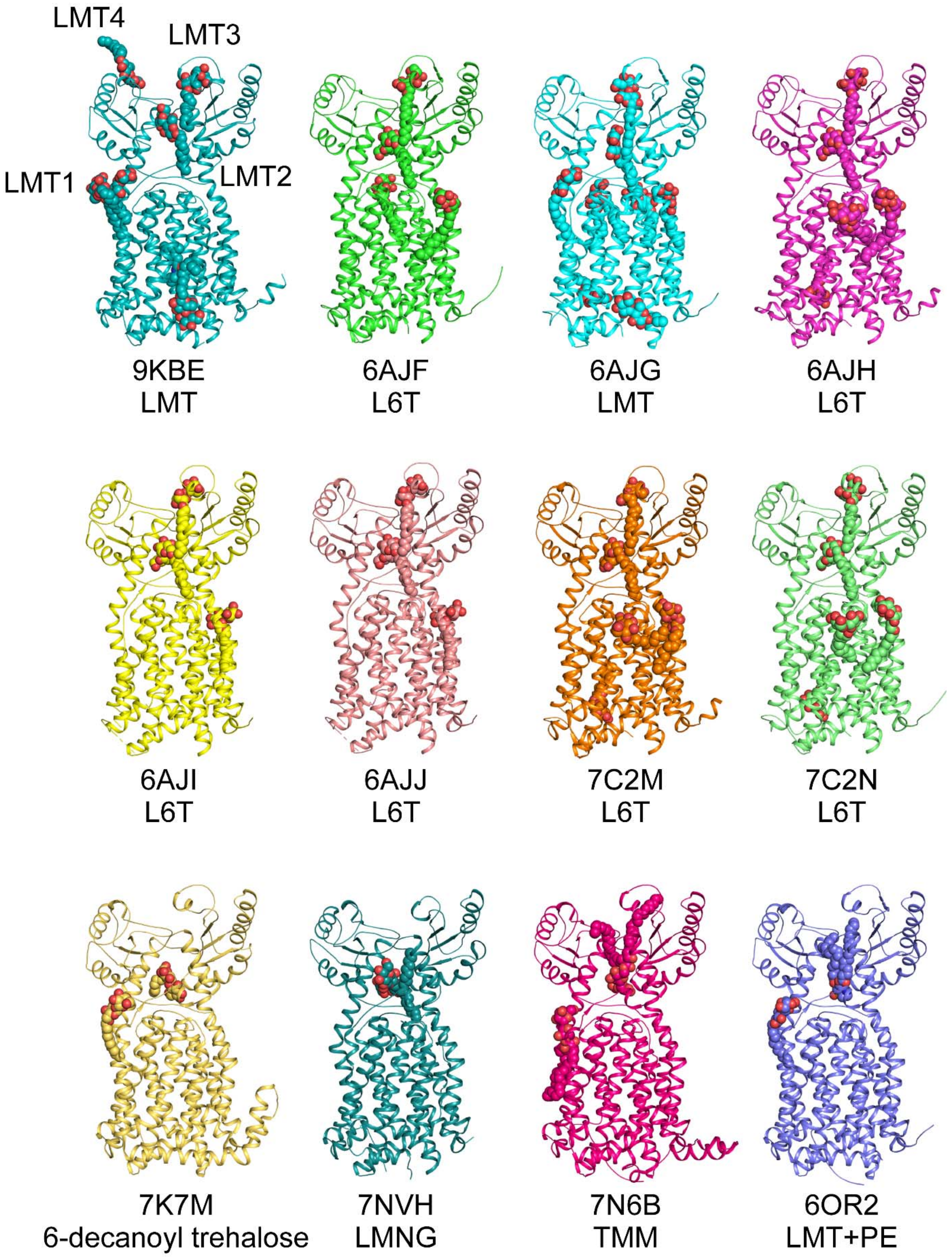

Figure S1 Cartoon representation of the reported MmpL3 structures. Bound substrate (TMM; Trehalose Monomycolate), lipid (PE; phosphatidylethanolamine) and detergents (LMT, dodecyl-β-D-maltoside, L6T; Lauryl-6-trehaloside, LMNG; Lauryl maltose neopentyl glycol, 6-decanoyl trehalose) are shown in CPK representation. Inhibitors have been deleted on this figure.

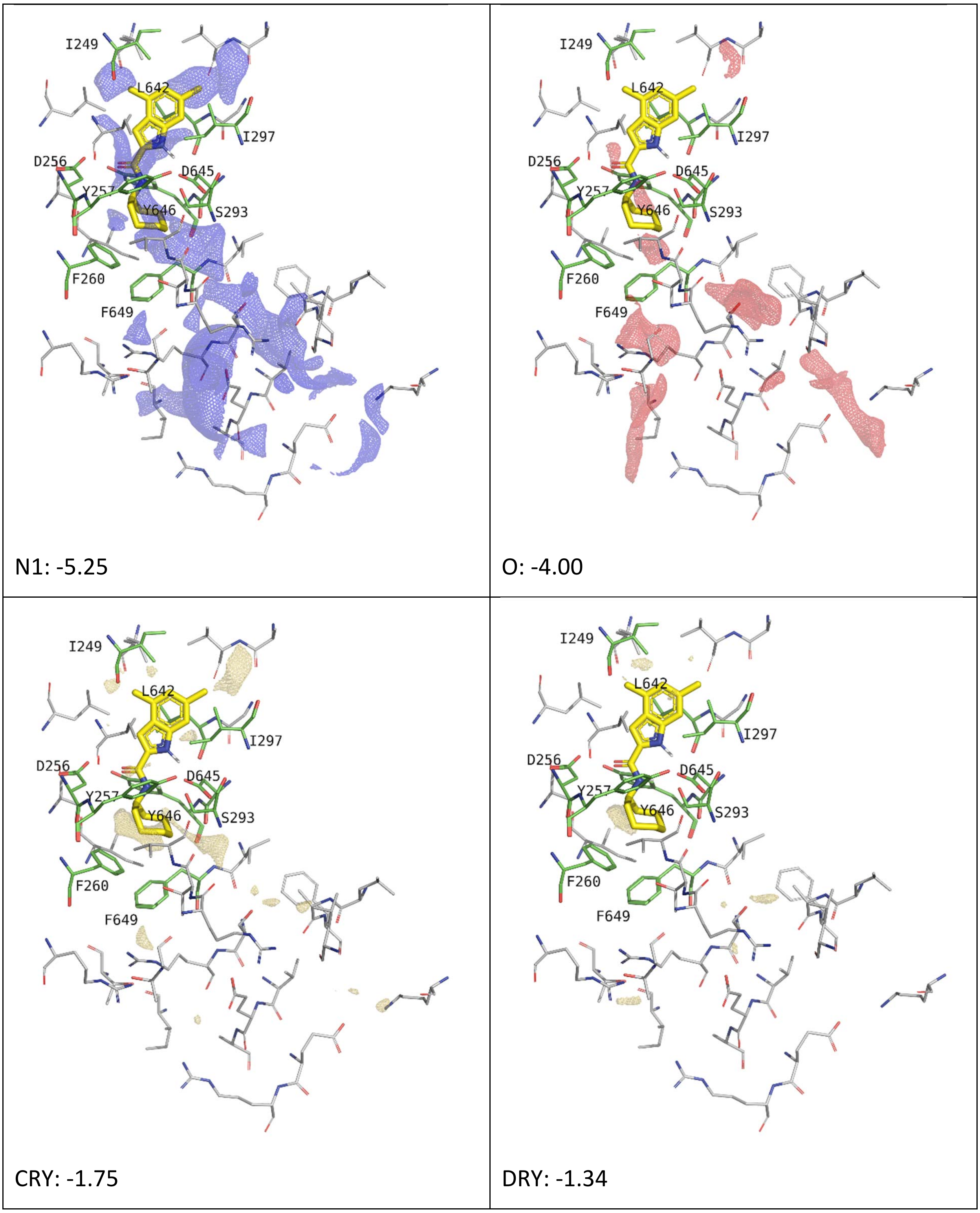

Figure S2 GRID hotspots obtained with probes N1, O, CRY and DRY. For each probe we report the energy value used to show the iso-contour surfaces.

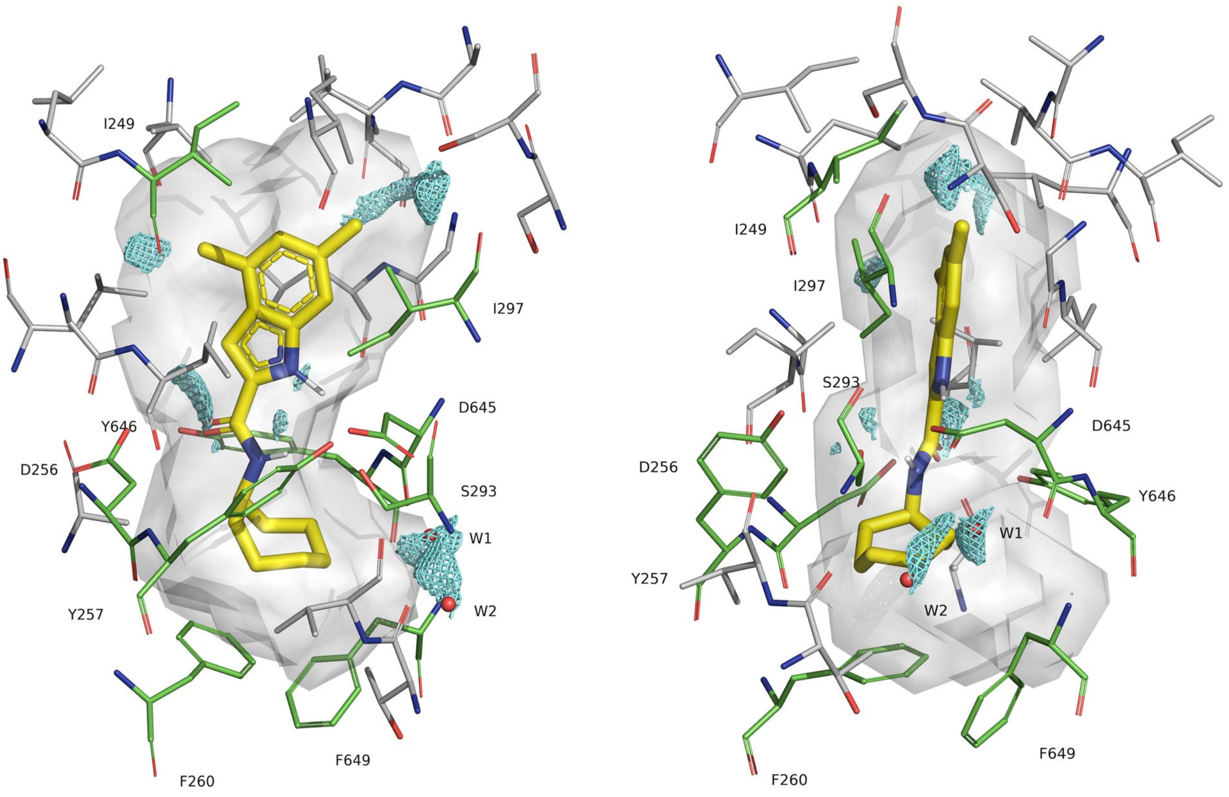

Figure S3 GRID hotspots obtained with probe OH2. Two different orientations for the UPAR-1109 in its binding pocket, with the two water molecules well aligned with the MIF from GRID (Energy values used of the MIF: H_2O *probe*= -8.00 kcal/mol)

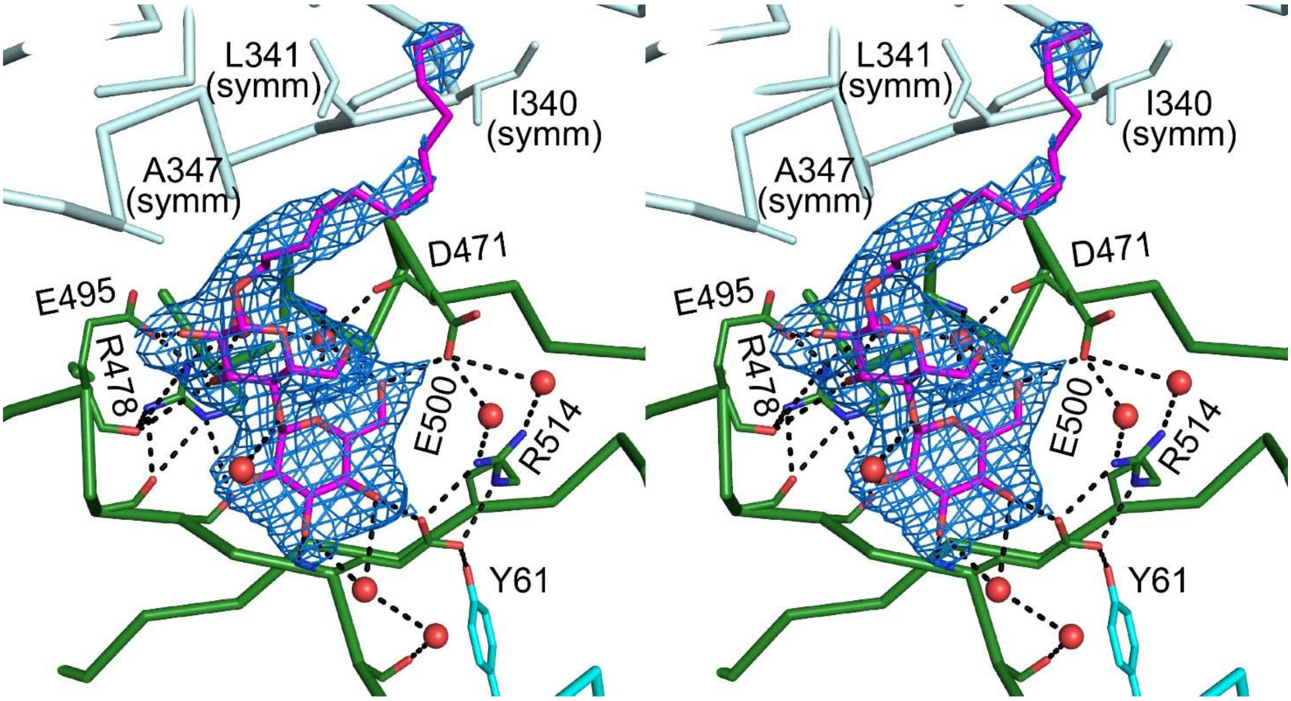

Figure S4 Figure 4A in stereo-pairs (A), and the 2*fo-fc* electron density map of LMT at 0.65 σ (B).

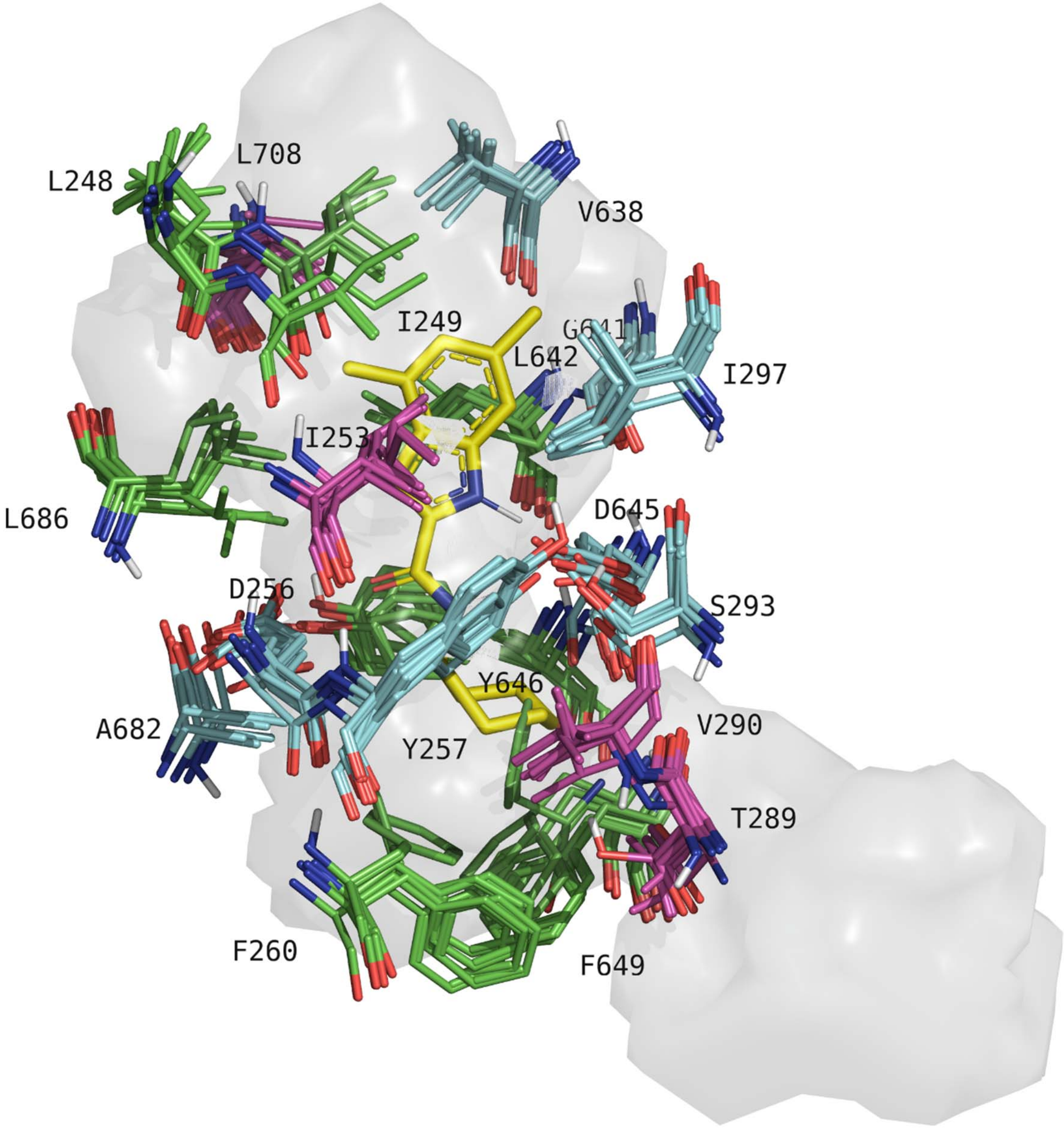

Figure S5 Alignment of the binding pocket residues, from the eight X-ray structures, to highlight the residues’ flexibility.

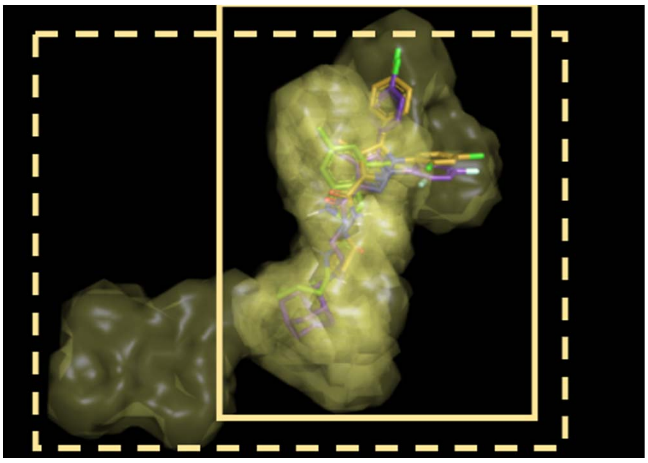

Figure S6 Scheme for the “large grid” (dashed line) and “small grid”.

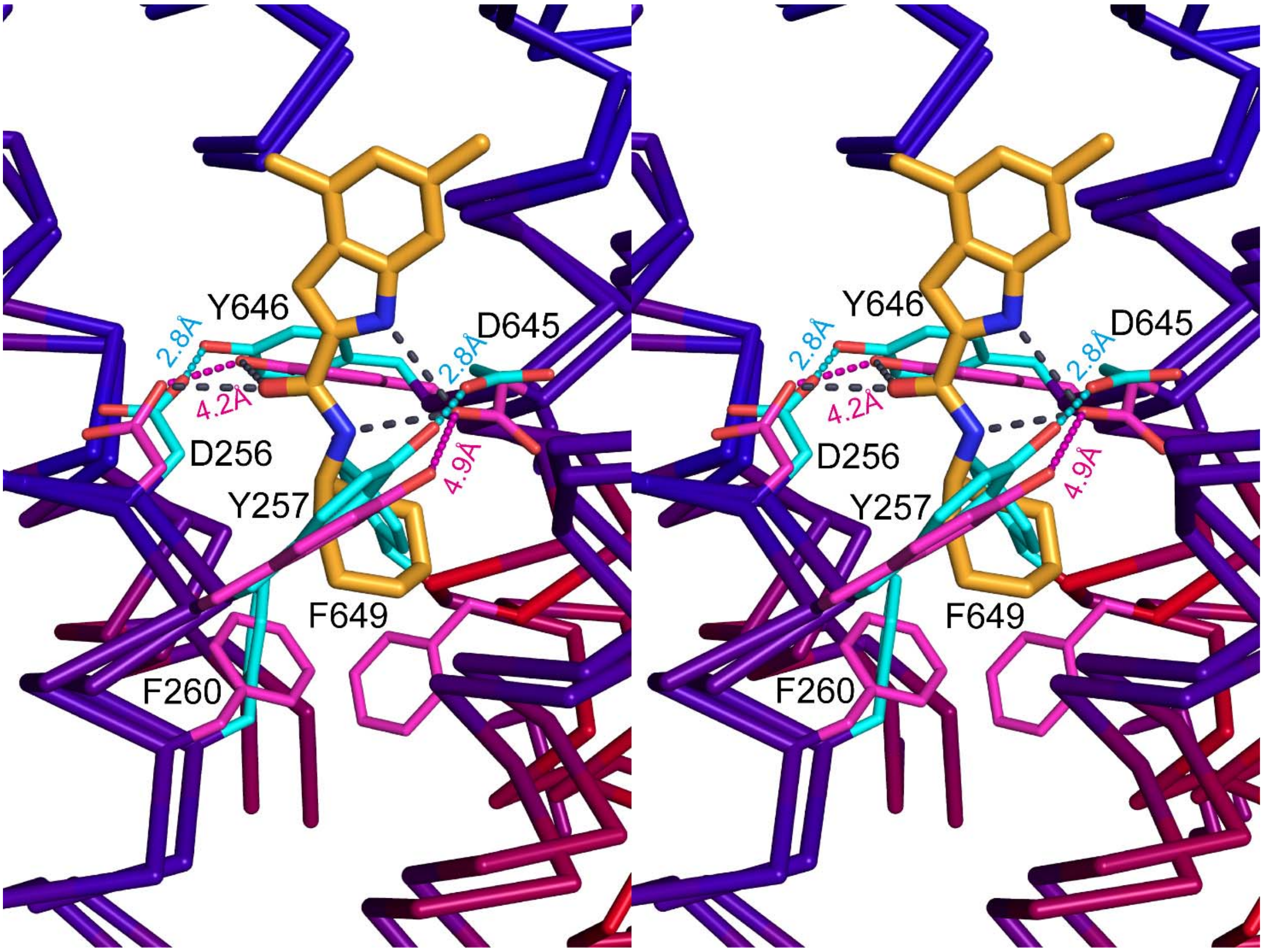

Figure S7 Stereo-pair version of Figure 3B.

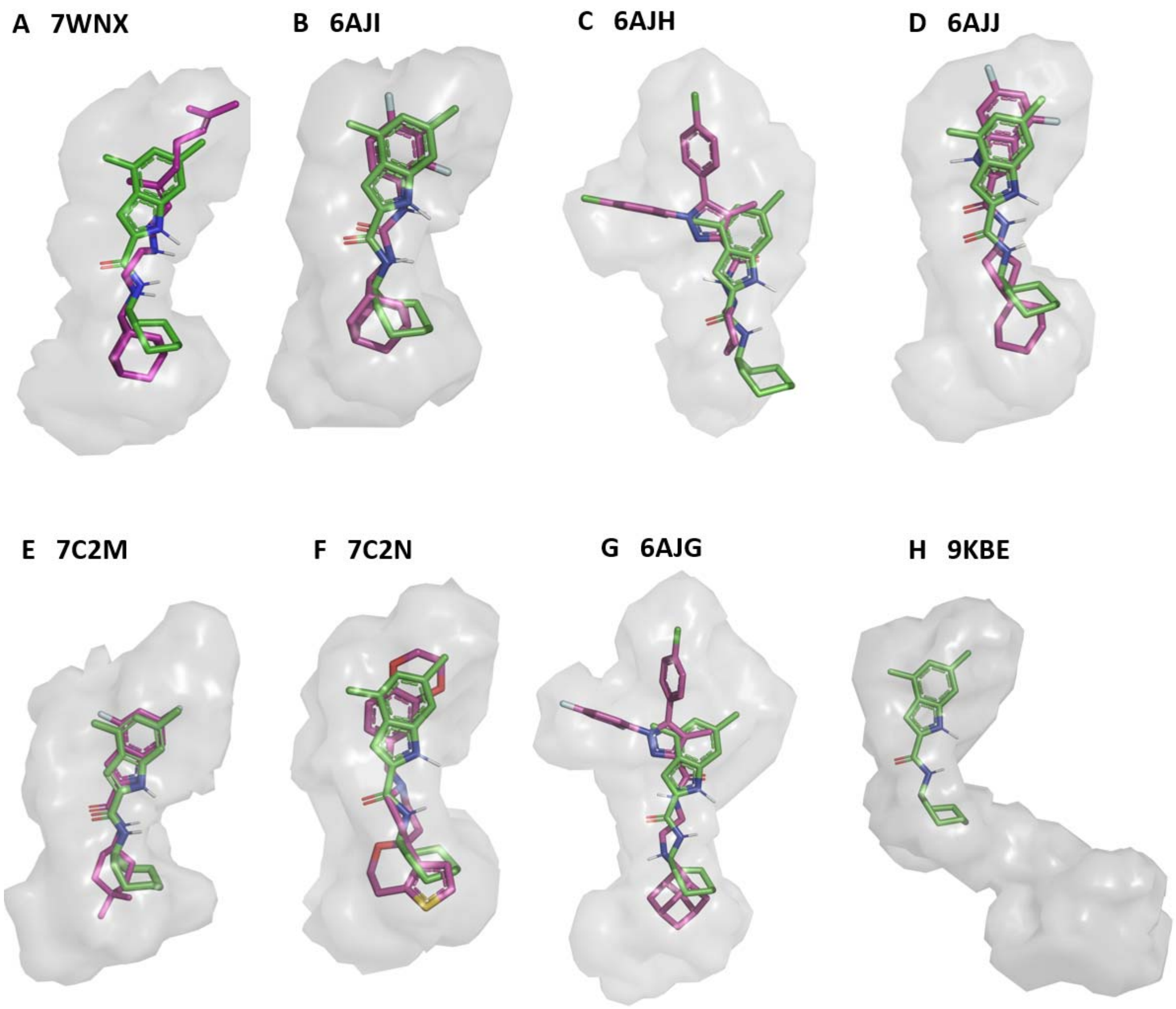

Figure S8 . Collection of the TM pocket of MmpL3 (in gray) with the ligand in the crystallographic structure (in pink) and UPAR1109 as a reference (in green).

Table S1 Comparison between residues in 7NVH (*M.tubercolosis*) and 9KBE (*M.smegmatis*).

**Residue in 7NVH (*M.tubercolosis*)**	**Residue in 9KBE (*M.smegmatis*)**	**Interactions**
I244	I249	Hydrophobic contact
I248	I253	Hydrophobic contact
Y252	Y257	Polar interaction
D251	D256	Hydrogen bond
F255	F260	Hydrophobic contact/ π- π stacking
L633	V638	Hydrophobic contact
I292	I297	Hydrophobic contact
G636	G641	Hydrophobic contact
L637	L642	Hydrophobic contact
S288	S293	Hydrophobic contact/ π- π stacking
D640	D645	Hydrogen bond
Y641	Y646	Polar interaction
F644	F649	Hydrophobic contact/ π- π stacking
T284	T289	Polar interaction/ hydrogen bond
V285	V290	Hydrophobic contact
V681	L686	Hydrophobic contact

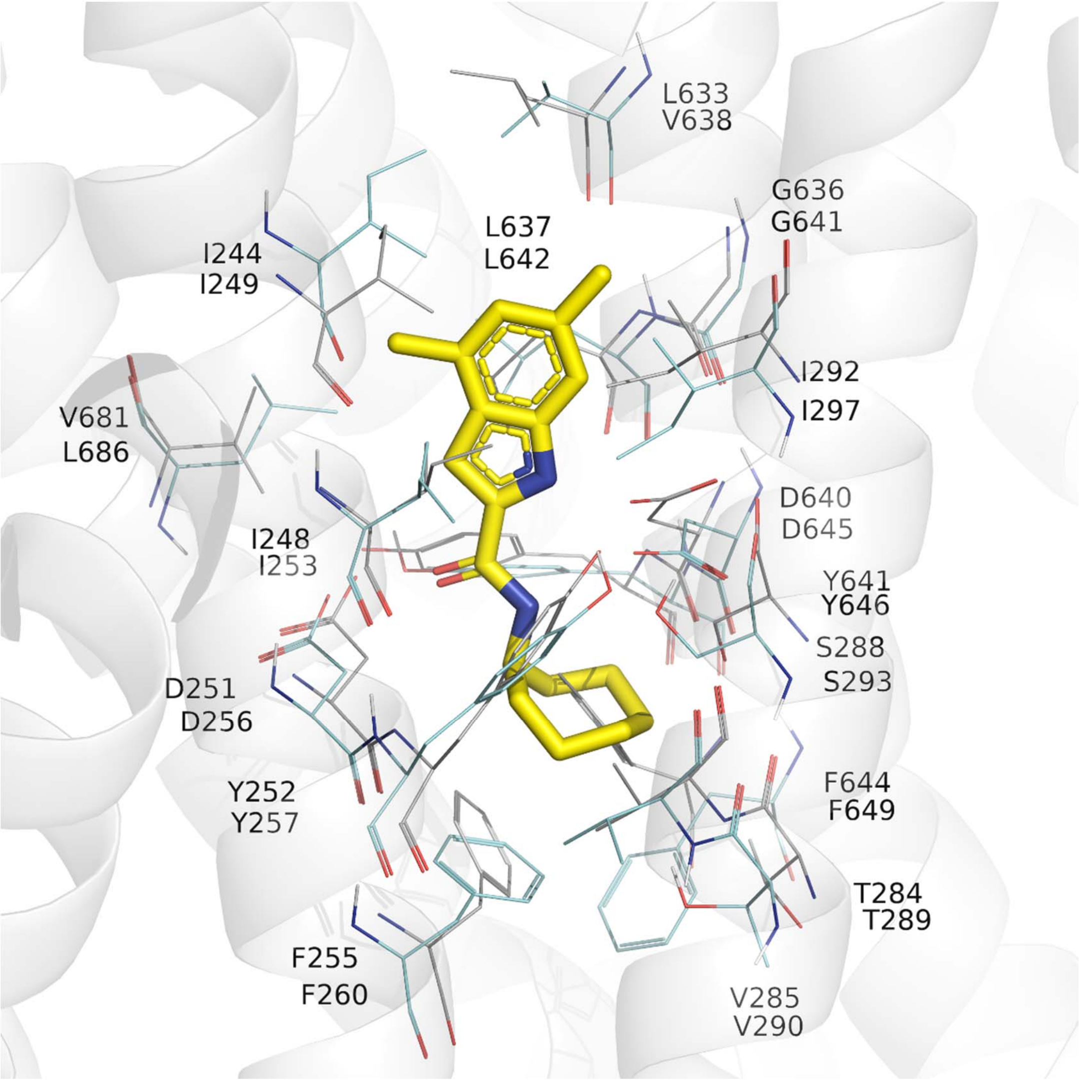

Figure S9 Superposition of the residues of the binding sites of 9KBE (*M.smegmatis* , in cyan 7NVH (*M.tubercolosis*, in grey); UPAR-1109 is used as reference (in yellow).

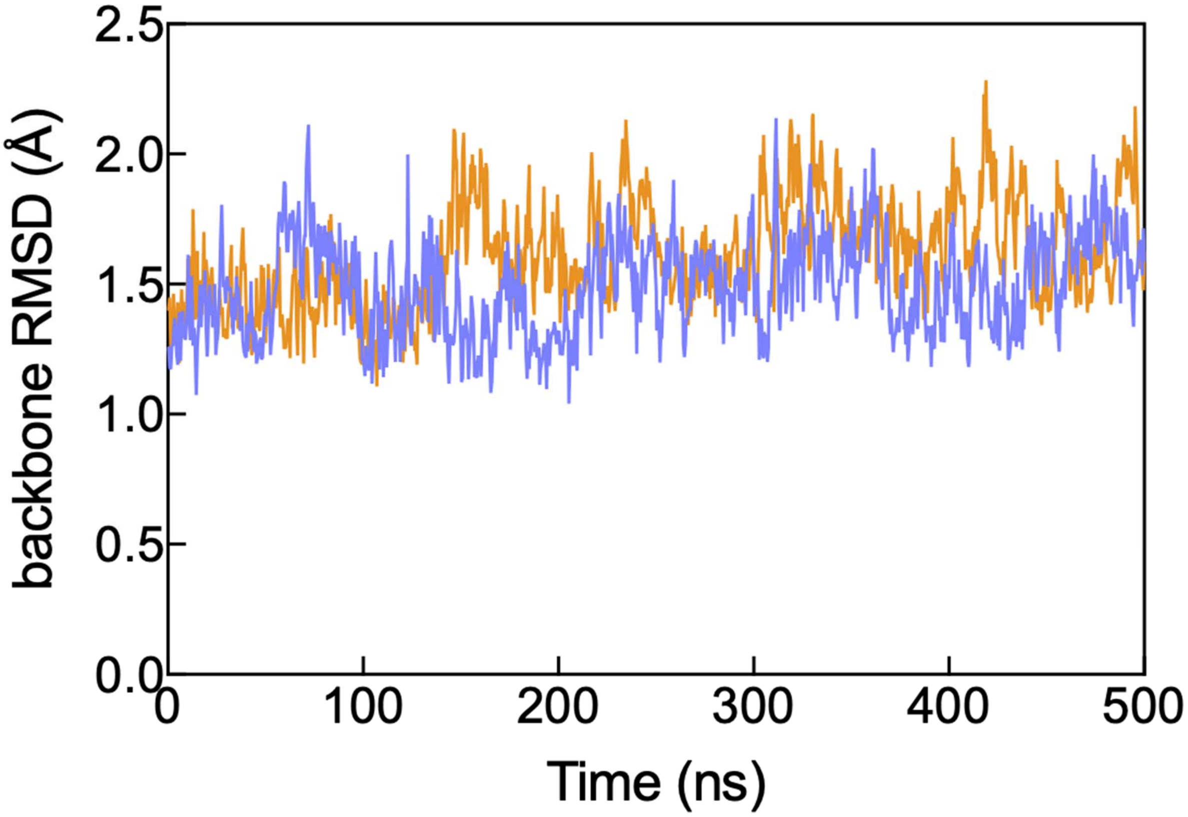

Figure S10 Backbone RMSD (relative to the crystallographic structure) vs time for the 500 ns unbiased simulations.
